# Machine Learning and Deep Learning Models for Predicting Future Falls in Community-Dwelling Older Adults: Systematic Review and Meta-Analysis of Longitudinal Evidence

**DOI:** 10.2196/84844

**Published:** 2026-05-14

**Authors:** Ying Gao, Doudou Xu, Xinru Li, Jue Wang, Linbin Wang, Beiwen Wu, Haifeng Zhao, Xian Qiu, Weiyi Zhu

**Affiliations:** 1 Department of Nursing, Ruijin Hospital, Shanghai Jiao Tong University School of Medicine, No.197, 2nd Ruijin Rd, Huangpu District, Shanghai, 200025, China, 86 02164370045; 2 School of Nursing, Shanghai Jiao Tong University School of Medicine, Shanghai, China; 3 Department of Orthopedics, Ruijin Hospital, Shanghai Jiao Tong University School of Medicine, Shanghai, China; 4 Department of Neurosurgery, Ruijin Hospital, Shanghai Jiao Tong University School of Medicine, Shanghai, China; 5 Department of Psychiatry, University of Cambridge, Cambridge, United Kingdom; 6 Institute of Medical Artificial Intelligence, Shanghai Jiao Tong University School of Medicine, Shanghai, China

**Keywords:** machine learning, falls, risk prediction, community-dwelling older adults, systematic review, meta-analysis

## Abstract

**Background:**

Machine learning (ML) and deep learning (DL) show promise for fall risk prediction, but prior reviews focused mainly on real-time fall detection, in-hospital falls, or conventional statistical models. The performance of ML-DL–based models for predicting future falls in community-dwelling older adults remains unclear.

**Objective:**

This study aimed to review ML-DL studies for predicting future falls among community-dwelling older adults and meta-analyze discrimination where feasible.

**Methods:**

Six databases were searched from inception to September 23, 2024, with updates on August 31, 2025, and February 28, 2026. We included longitudinal studies developing or validating ML-DL models to predict future falls in community-dwelling adults aged ≥60 years and excluded real-time detection, simulated or no fall, and inpatient studies. Risk of bias was assessed using the Prediction Model Risk of Bias Assessment Tool (PROBAST). Areas under the curve (AUCs) were meta-analyzed using Hartung-Knapp-Sidik-Jonkman random-effects models with 95% CIs. Heterogeneity, 95% prediction intervals (PIs), sensitivity analyses, and subgroup analyses were conducted.

**Results:**

After screening 10,253 records, 28 (0.3%) studies were included; 18 (64.3%) focused on general older adults. Prediction horizons ranged from 3 months to 7 years, and fall incidence ranged from 1.6% to 46.6%. Twenty-three (82.1%) studies applied ML, and 5 (17.9%) studies used DL. Input modalities included text (n=18, 64.3%), sensor (n=5, 17.9%), image (n=1, 3.6%), and multimodal data (n=4, 14.3%). Common predictors included age, sex, fall history, depression, and basic daily activities. Only one model underwent external validation. Calibration reporting was sparse. All models were rated at high risk of bias. Ten models were meta-analyzed, yielding a pooled AUC of 0.79 (95% CI 0.69‐0.87) with extreme heterogeneity (*τ*^2^=0.64; *τ*=0.80; *I*^2^=99.8%; *Q*=4128.99). The confidence-distribution bootstrap PI was 0.20 to 0.99, indicating substantial uncertainty in expected performance across new populations. Subgroup analyses indicated moderation by sample size and population type, with higher discrimination in specific populations than in general samples; however, the specific population subgroup included only 2 studies. Although all participants were community dwelling, some cohorts were recruited through clinically enriched pathways rather than general community sampling.

**Conclusions:**

ML-DL models show potential for identifying community-dwelling older adults at elevated future fall risk; however, wide PIs, limited external validation, and high risk of bias suggest real-world performance may be optimistic. The pooled AUC should be interpreted as a summary of reported discrimination under study-specific conditions, predominantly from internally validated, high-risk-of-bias models, rather than as a robust estimate of transportable real-world performance. This review extends prior reviews by focusing on community-dwelling settings and by integrating PROBAST, Hartung-Knapp-Sidik-Jonkman meta-analysis, PIs, and modality-specific synthesis to evaluate both discrimination and uncertainty. Findings support the use of ML-DL models for proactive fall prevention while emphasizing the need for validation and context-specific implementation.

## Introduction

### Rationale

Falls, defined by the World Health Organization as events in which an individual unintentionally comes to rest on the ground, floor, or another lower level [1,2], are among the most common and serious health concerns in community-dwelling older adults. Approximately 26% of older adults worldwide experience at least one fall each year [2,3]. In the United States, more than 25% of community-dwelling older adults fall annually, and 37% of these incidents result in injuries requiring medical care [4,5]. Common adverse outcomes include fractures, head injuries, functional decline, mobility limitations, and premature institutionalization [6-8], placing a heavy burden on caregivers and community health care systems. Globally, falls account for approximately 20% to 30% of moderate to severe injuries among older adults and represent the second leading cause of injury-related death [9]. Beyond physical consequences, falls are also associated with psychological distress, including depression and fear of falling, which can substantially reduce quality of life and independence [10,11]. With the rapid aging of populations worldwide, both the incidence and consequences of falls among older adults are expected to increase further [2].

Unlike institutionalized older adults, who receive structured supervision and continuous monitoring from health care providers, community-dwelling older adults often have limited access to intensive monitoring or care services [12]. Consequently, individuals at high risk of falling may remain unidentified until an adverse event occurs. Several clinical assessment tools have been developed to identify individuals at risk of falls, including the Timed Up and Go test [13], Berg Balance Scale [14], and various multifactorial fall risk assessment instruments. However, these tools typically rely on a limited set of predefined functional or clinical indicators, which are most commonly measures of mobility, balance, or physical performance, and may not fully capture the complex and multifactorial determinants of falls in community-dwelling older adults [8]. In addition, although wearable sensor technologies have been increasingly developed and can facilitate rapid responses after a fall occurs, most focus on real-time fall detection rather than prospective fall risk prediction [15]. Thus, effective early identification of high-risk individuals in community and home settings, before a fall occurs, remains a critical challenge for implementing timely and targeted prevention strategies.

Traditional statistical approaches, such as logistic regression models, have commonly been used to estimate fall risk based on predefined predictors [16]. While these models offer interpretability and clinical familiarity, they are often limited in their ability to model nonlinear relationships, high-dimensional predictors, and complex associations among risk factors [17-19]. This is particularly problematic in community populations, where fall risk is influenced by a wide range of interrelated factors, including physical function, psychological conditions, lifestyle behaviors, environmental hazards, and social support [20,21].

Recent studies have identified numerous predictors for falls, including age [22,23], history of falls [24,25], comorbidities [26,27], use of high-risk medications [22], functional performance (eg, activities of daily living [ADLs] or instrumental activities of daily living [24,28]), gait and balance deficits [29], cognitive impairment, and psychological factors such as fear of falling [30]. These findings highlight the multifactorial and context-dependent nature of falls in community settings, where diverse health, functional, and social factors jointly influence fall risk. As a result, conventional regression-based models may oversimplify these relationships and exhibit limited predictive performance when applied to heterogeneous real-world populations.

In this context, machine learning (ML) methods, including deep learning (DL), offer a promising alternative for improving fall risk prediction and supporting individualized prevention by integrating heterogeneous predictors, accommodating nonlinear relationships, and leveraging multimodal data sources [31]. ML approaches such as decision trees and random forests (RFs) learn predictive rules directly from data without requiring prespecified functional forms, and they often provide relatively transparent model structures or feature-importance measures that can facilitate clinical interpretation [32]. DL, as a subfield of ML, uses multilayer neural networks to learn hierarchical representations from raw or minimally processed inputs, which can be advantageous for high-dimensional modalities such as wearable sensor time series and imaging data [33,34]. Both ML and DL are important: ML remains a pragmatic and interpretable baseline for many structured clinical and survey datasets, whereas DL may be better suited to complex signals but typically requires larger sample sizes and more explicit strategies to ensure interpretability and generalizability in medical settings.

Although the importance of fall risk management and the potential of ML have been widely recognized, existing reviews focused on institutionalized real-time fall detection, in-hospital fall risk prediction, or conventional statistical models, rather than ML-based models designed to predict future falls for primary prevention and early identification among the community-dwelling older adults. For example, a 2025 review by Xie et al [35] focused on ML-based models for short-term in-hospital fall outcomes, limiting their applicability to community settings where risk profiles differ substantially. A 2024 review by Dormosh et al [36] examining community-dwelling older adults primarily evaluated traditional statistical prediction models, with limited emphasis on ML-DL approaches. In addition, reviews by Usmani et al [18] and Ren et al [37] focused on real-time or near-real-time fall detection using sensor-based data in controlled or laboratory settings, rather than prospective risk prediction in real-world environments, thereby limiting their relevance for primary prevention and early risk identification in community-dwelling populations. To date, no systematic review has comprehensively evaluated the predictive performance, methodological quality, and clinical applicability of ML-based fall risk prediction models specifically designed to predict future falls among community-dwelling older adults for primary prevention and early risk identification.

### Objectives

To address these gaps, this systematic review and meta-analysis aims to (1) systematically summarize ML- and DL-based prediction models for future falls in community-dwelling older adults and evaluate their predictive performance; (2) assess the methodological quality and risk of bias of included models; and (3) synthesize discrimination performance across studies using meta-analysis of area under the curve (AUC) values, and explore potential sources of heterogeneity through subgroup analyses and meta-regression, to inform the development and implementation of robust, evidence-based tools for fall prevention in real-world community settings.

## Methods

### Protocol and Registration

This systematic review and meta-analysis was registered in PROSPERO under the registration number CRD42024580902. Deviations from the registered protocol should be noted. First, the literature search was updated to include studies published up to February 28, 2026. Second, the final meta-analysis was restricted to studies reporting the AUC with corresponding 95% CIs, focusing on model discrimination performance. Third, because all included models were judged to be at high risk of bias, the prespecified sensitivity analysis excluding high-risk studies was not feasible; instead, leave-one-out analyses and additional sensitivity analyses addressing potential overlap among the China Health and Retirement Longitudinal Study (CHARLS)–derived cohorts were performed. Additional statistical methods were refined during the analysis to improve methodological rigor.

The review was reported in accordance with the PRISMA (Preferred Reporting Items for Systematic Reviews and Meta-Analyses) 2020 statement [38], and the complete PRISMA 2020 expanded checklist [38] is provided in Table S1 in Checklist 1. To strengthen transparency, the search and its reporting followed the PRISMA-S (PRISMA literature search extension) [39] (Table S2 in Checklist 1). As the review synthesized model discrimination (AUC) across studies, selected elements of the PRISMA-DTA (PRISMA Statement for Diagnostic Test Accuracy) guideline were additionally consulted to inform the reporting structure where applicable [40], and the corresponding checklist is provided in Table S3 in Checklist 1.

### Eligibility Criteria

Studies were included if they met the following criteria: (1) community-dwelling older adults aged 60 years or older at baseline; (2) longitudinal studies, cohort studies, or case-control studies; (3) predictors measured at baseline; (4) ML models, including DL, for identifying participants at high risk of future falls; (5) assessment of fall-related outcomes, such as fall occurrence, fall rate, number of fallers, or fall risk; and (6) publication in English with full-text available.

Studies were excluded based on the following criteria: (1) detection of real-time falls; (2) no actual falls, such as simulated falls, fear of falling, abnormal gait, and mobility decline; (3) inpatient falls within hospital or institutional settings; and (4) reviews, meta-analyses, editorials, letters, study protocols, case reports, abstracts, preprints, or other forms of secondary research.

### Information Sources

We conducted a comprehensive literature search across 6 databases, including PubMed, Embase, Web of Science Core Collection, CINAHL, Cochrane Library, and IEEE Xplore, from their inception to September 23, 2024. The search was subsequently updated on August 31, 2025, and February 28, 2026, using the same search strategies. Additionally, reference lists of included studies were examined to identify potentially eligible articles. Clinical trial registries were not searched because this review focused on published prediction model studies. No additional online resources, conference proceedings, or website browsing were systematically searched. Study authors or experts were not contacted to identify additional studies or obtain unpublished data. No additional search methods beyond electronic database searching and reference list screening were used.

### Search

The search strategy combined controlled vocabulary terms (eg, MeSH and Emtree) such as “aged,” “artificial intelligence,” “machine learning,” and “accidental fall,” with relevant free-text terms. No limits or restrictions were applied during the literature search to maximize inclusivity. No published search filters were used. The detailed search strategies for all databases are provided in Multimedia Appendix 1. No previously published search strategies were adopted or adapted. The search strategies were peer reviewed by an information specialist prior to implementation.

### Study Selection

Duplicate records were removed using Covidence [41] prior to screening. The same platform was used to manage the study screening process. After removing duplicate studies, 3 authors (YG, DX, and XL) independently screened the remaining articles in pairs based on titles and abstracts to assess their eligibility. Due to language accessibility, only studies published in English were included at the eligibility stage. Following the initial screening, full-text reviews were conducted for the retained articles to finalize their inclusion, and the reference lists of included studies were then manually examined to identify any potentially relevant research. Any discrepancy among reviewers was resolved through consultation with a fourth author (XQ).

### Data Collection Process

Data extraction was performed independently by 3 reviewers (YG, DX, and XL) using a standardized extraction form. All extracted data were cross-checked by 1 reviewer (YG) to ensure accuracy and consistency, and discrepancies were resolved by discussion with a fourth reviewer (XQ).

### Definitions for Data Extraction

Data extraction consisted of two parts: (1) basic study characteristics, including author, country, year of publication, outcome time perspective, study design, years of data collection, data source, single-center or multicenter design, sampling method, group, sample size, participant characteristics, outcome and definition, whether the outcome was first-time fall, number of fallers, and follow-up duration; and (2) model-related information, including methods for handling missing values, data-splitting strategy, feature selection methods, modeling techniques, final predictors used in the model, calibration, AUC, and other performance metrics.

### Risk of Bias and Applicability

To assess the risk of bias and applicability of the included studies, the Prediction Model Risk of Bias Assessment Tool (PROBAST) was used [42]. PROBAST consists of 20 signaling questions across 4 domains: participants, predictors, outcome, and analysis. Each question can be answered as “yes,” “probably yes,” “no,” “probably no,” or “no information,” where responses of “yes” or “probably yes” indicate low risk of bias, and “no” or “probably no” indicate high risk of bias. A domain is judged to be at high risk of bias if at least one signaling question is rated as “no” or “probably no.” The overall risk of bias is considered low only when all domains are judged to have low risk. Three reviewers (YG, DX, and XL) independently evaluated the risk of bias and concerns regarding applicability using the PROBAST checklist. Discrepancies between reviewers were resolved through discussion.

### Prediction Performance Measures

The primary performance metric extracted from each study was the AUC, which reflects the discrimination ability of prediction models for identifying community-dwelling older adults at risk of future falls. When available, additional performance metrics, such as accuracy, sensitivity, specificity, and calibration measures, were also recorded.

### Synthesis of Results

All statistical analyses were conducted in R software (version 4.5.2; R Foundation for Statistical Computing) using the metafor package (version 4.8‐0 [43]), meta package (version 8.2-1 [44]), and pimeta package (version 1.1.3 [45]).

Studies were eligible for quantitative synthesis if they reported AUC values with corresponding 95% CIs. Studies that did not report AUC values or corresponding 95% CIs were excluded. As AUC is a bounded measure (0‐1), pooling was conducted on the logit-transformed scale to stabilize variances and improve the normality assumption. For each model, SEs of the logit-transformed AUC were derived from the reported 95% CIs. Specifically, the lower and upper limits of the reported CI were first transformed using the logit function. Under the assumption that the logit-transformed AUC follows an approximately normal distribution, the SE on the logit scale was then estimated as (logit upper limit−logit lower limit)/(2×1.96). This approach derives the SE directly on the transformed scale and avoids reliance on first-order delta method approximations, thereby better accounting for the nonlinear nature of the logit transformation. Pooled estimates were subsequently back-transformed to the original AUC scale using the inverse-logit function for interpretation. In total, 9 studies comprising 10 independent cohorts and their corresponding prediction models evaluated using internal validation were included in the quantitative synthesis. The only model evaluated using external validation, along with several other models, was excluded due to insufficient information to derive SEs.

Between-study heterogeneity was quantified using *I^2^*, τ^2^, τ, and *Q* statistic using the metafor package. Although *I^2^* values of 25%, 50%, and 75% are commonly interpreted as low, moderate, and high heterogeneity [46], respectively, *I^2^* alone does not convey the magnitude of dispersion across different populations or settings [47]. τ^2^ represents the variance of true effects across studies, and τ represents the corresponding SD. The *Q* statistic (Cochran *Q* test) was used to formally assess whether observed variability in effect estimates exceeded that expected by chance, with a significant value indicating the presence of heterogeneity.

To estimate the expected range of model performance in future populations and better quantify the real-world implications of heterogeneity, 95% prediction intervals (PIs) were additionally calculated using the pimeta package. PIs reflect the variation in true effects across settings and indicate the range of effects that may be expected in a future study [48]. Four PI estimation methods were applied, including the Higgins-Thompson-Spiegelhalter (HTS) method, the Hartung-Knapp–adjusted method (HTS-HK), the Sidik-Jonkman method (HTS-SJ), and the confidence-distribution bootstrap method proposed by Nagashima et al [49]. As HTS-type PIs rely on plug-in estimation of the heterogeneity variance and large sample approximations, their coverage may be suboptimal when the number of studies is small. In contrast, the confidence-distribution bootstrap approach more fully accounts for uncertainty in τ^2^ and has been shown to achieve coverage closer to the nominal level in meta-analyses with few studies. Therefore, the confidence-distribution bootstrap PI was selected as the primary PI displayed in the main forest plots, whereas the other PI methods were retained as supplementary analyses.

As several prediction models were derived from the same large population-based database (CHARLS), particular attention was paid to the potential risk of double-counting when synthesizing model performance. Following methodological recommendations highlighting that duplicate inclusion of the same evidence may lead to overstating the precision of pooled estimates in meta-analysis, each model was examined to determine whether it represented a duplicated analysis of the same participants or a distinct analytic cohort or subpopulation [50]. Models derived from mutually exclusive subgroups or from different analytic cohorts with distinct modeling strategies were treated as separate analytical units.

### Meta-Analysis

The metafor package was used for effect estimation, while the meta package was used for visualization of forest plots. Random-effects meta-analyses were performed using the Sidik-Jonkman estimator for between-study variance (method=“SJ”), together with the Hartung-Knapp adjustment (test=“knha”), known as the Hartung-Knapp-Sidik-Jonkman (HKSJ) method [51]. This approach yields more accurate CIs, produces more stable pooled estimates, and reduces false-positive rates compared with the traditional DerSimonian-Laird method, particularly when heterogeneity is substantial or the number of studies is small [51].

The primary meta-analysis included the best-performing model from each study cohort, as is commonly done in prediction model syntheses. To account for studies that developed multiple models from the same cohort, a within-study weighted average meta-analysis was additionally conducted. For each study cohort, model-level AUCs were first transformed to the logit scale and then combined using inverse-variance weighting to obtain a single cohort-level average AUC. This approach mitigates the potential inflation of pooled performance that may arise when only the best-performing model from each study is considered. The resulting cohort-level averages were then used in a random-effects meta-analysis with the same HKSJ framework.

### Additional Analyses

Additional analyses included sensitivity analyses, assessments of potential small-study effects and publication bias, subgroup analyses, and meta-regression. All analyses were conducted using the metafor package.

Robustness was evaluated using leave-one-out sensitivity analyses, in which each study was sequentially excluded and the pooled effect recalculated. Additional predefined sensitivity analyses were conducted to evaluate the potential impact of correlated cohorts arising from the use of the CHARLS database. These analyses included sequential models retaining only one CHARLS-derived model at a time and an analysis excluding all CHARLS-derived models. Changes in pooled AUC estimates were examined to assess whether potential participant overlap materially influenced the overall results.

Potential small-study effects and publication bias were examined using funnel plots and Egger-type regression tests; the Begg test was not used due to its even lower statistical power in small samples. A nonsignificant *P* value from the Egger test indicates no strong evidence of funnel plot asymmetry; however, interpretation should be made with caution because both funnel plots and regression-based asymmetry tests have limited power and may be misleading when the number of studies is small [52]. Subgroup forest plots, leave-one-out influence plots, and funnel plots were generated using the meta package for visualization. A 2-tailed *P* value of <.05 was considered statistically significant.

Subgroup analyses were conducted using the metafor package, according to prediction time window (≤1 y vs >1 y), sample size (≤500 vs >500 participants), model modality (text-based vs sensor-based or multimodal inputs), and population subgroup (general vs specific conditions). Subgroup analyses by validation type (internal vs external), fall outcome definition (eg, first fall, multiple falls, or falls or syncope occurrence), model type (ML vs DL), and geographic region were not performed because these categories were represented by a single study or insufficient data were available for inclusion in quantitative synthesis. Within each subgroup, random-effects meta-analyses were performed using the same HKSJ framework, and subgroup-specific pooled AUCs with corresponding 95% CIs, heterogeneity statistics (*I^2^*, *τ*^2^, *τ*, and the *Q* statistic), and PIs were reported.

To formally assess differences between subgroups, mixed-effects meta-regression models were fitted, with subgroup indicators specified as categorical moderators. Between-study variance was estimated using the Sidik-Jonkman method, and statistical inference was based on the Hartung-Knapp adjustment. Differences between subgroups were evaluated using omnibus tests of moderators, and the proportion of between-study heterogeneity explained by each moderator was quantified using the *R*^2^ statistic.

## Results

### Study Selection

**Figure 1.**
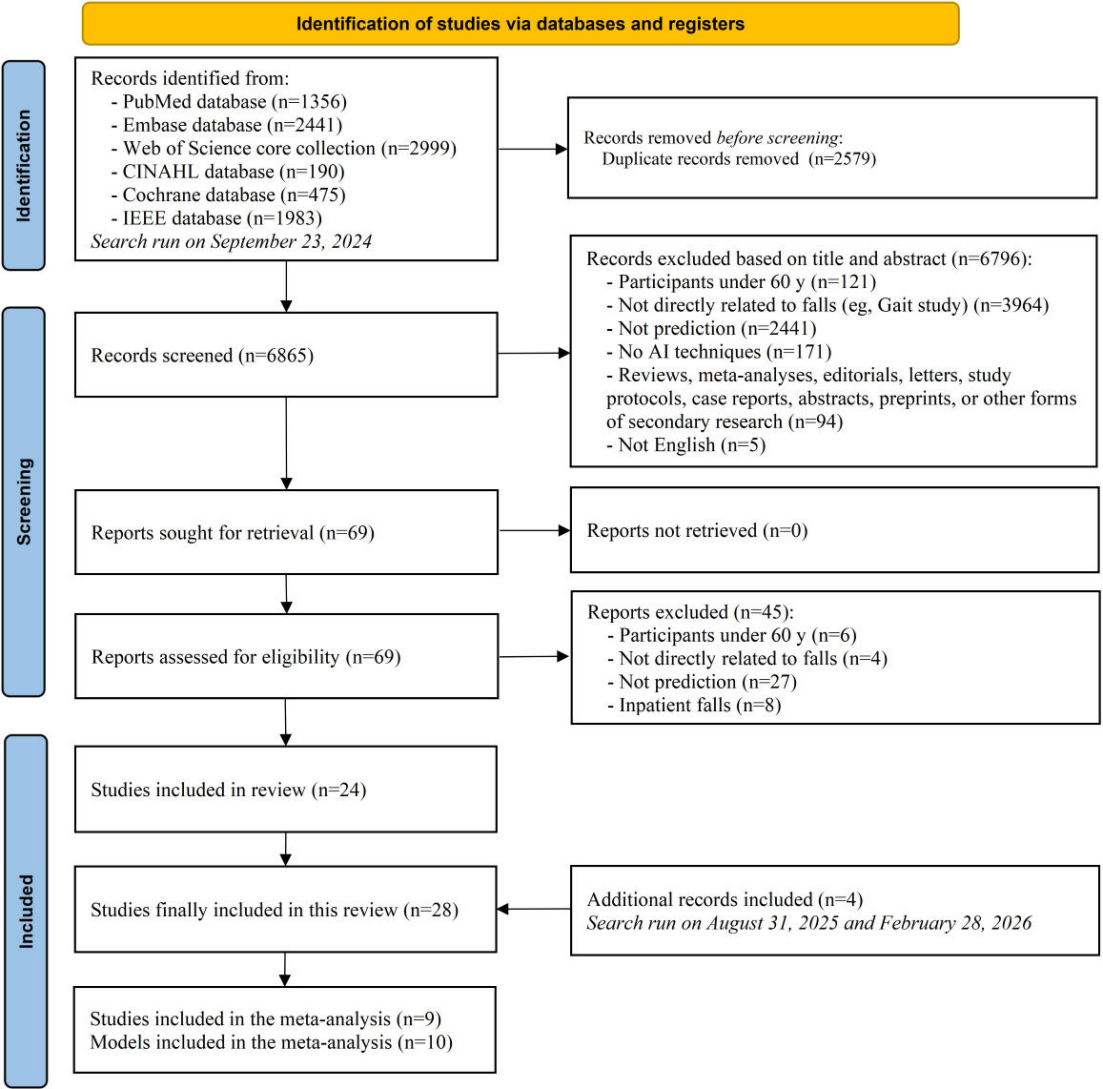
PRISMA (Preferred Reporting Items for Systematic Reviews and Meta-Analyses) flowchart illustrating the identification, screening, eligibility assessment, and inclusion of longitudinal studies evaluating machine learning and deep learning models for predicting future falls among community-dwelling older adults. Searches were performed across PubMed, Embase, Web of Science Core Collection, CINAHL, Cochrane Library, and IEEE Xplore databases from inception to September 23, 2024, and updated on August 31, 2025, and February 28, 2026. AI: artificial intelligence.

Figure 1 summarizes the search and selection process. After removing duplicates, 6865 records were identified. Following title and abstract screening, 6796 (98.6%) articles were excluded. Of the 69 (1%) full-text articles assessed, 45 (65.2%) did not meet the inclusion criteria, such as focusing on real-time fall detection or inpatient falls, and were excluded. Updated searches were conducted on August 31, 2025, and February 28, 2026, which identified an additional 3388 records. After screening and eligibility assessment, 4 additional studies met the inclusion criteria. Ultimately, a total of 28 studies were included in this review, and 9 studies, including 10 models, were included in the meta-analysis.

### Study Characteristics

**Figure 2.**
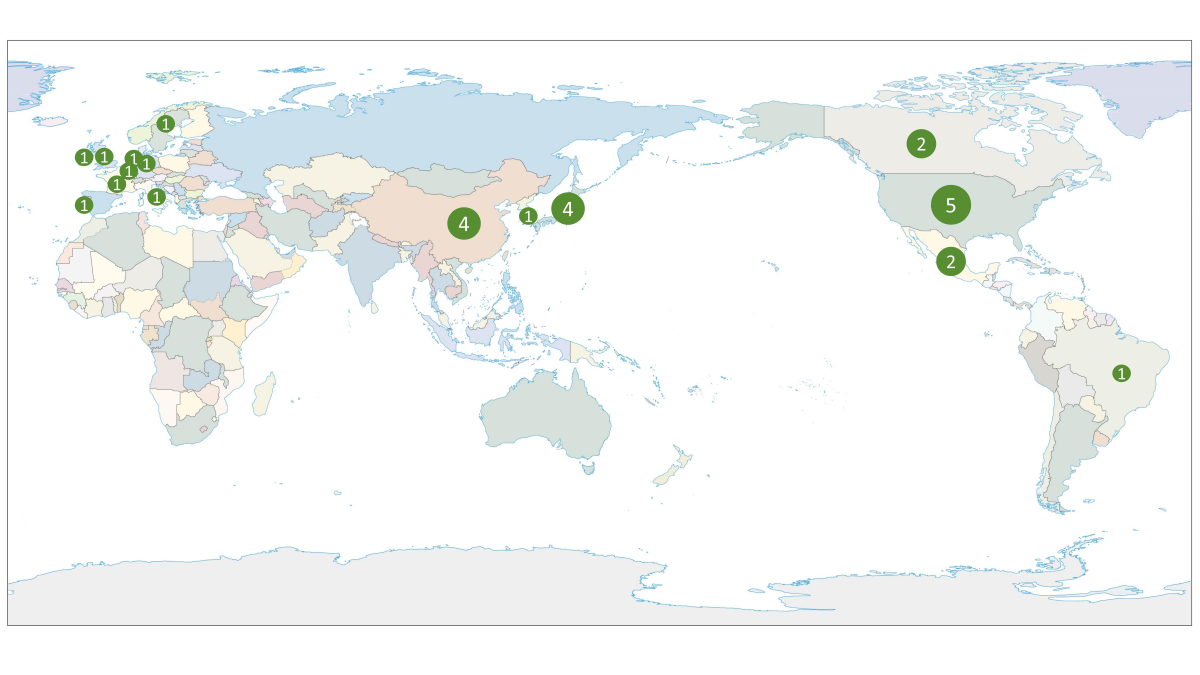
Geographic distribution of included studies. This figure illustrates the geographic locations of participant recruitment in the included longitudinal studies, with participant data originating from the United States (n=5), China (n=4), Japan (n=4), Canada (n=2), Mexico (n=2), Belgium (n=1), France (n=1), Germany (n=1), Brazil (n=1), Ireland (n=1), Italy (n=1), the Netherlands (n=1), Portugal (n=1), Sweden (n=1), the United Kingdom (n=1), and South Korea (n=1), indicating that most studies were conducted in East Asia, North America, and Europe, while other global regions remain underrepresented.

Table 1 summarizes the study design and participant characteristics of the 28 included studies. These studies were published between 2000 and 2026, with data originating from the United States (n=5, 17.9%), China (n=4, 14.3%), Japan (n=4, 14.3%), Canada (n=2, 7.1%), Mexico (n=2, 7.1%), Belgium (n=1, 3.6%), France (n=1, 3.6%), Germany (n=1, 3.6%), Brazil (n=1, 3.6%), Ireland (n=1, 3.6%), Italy (n=1, 3.6%), the Netherlands (n=1, 3.6%), Portugal (n=1, 3.6%), Sweden (n=1, 3.6%), the United Kingdom (n=1, 3.6%), and South Korea (n=1, 3.6%), concentrated in East Asia, North America, and Europe. The remaining regions are underrepresented (Figure 2).

**Table 1.** Summary of study design and participant characteristics.

Author (year)	Country	Study design (collection period)	Data source	Single- or multi-center (number, if multicenter)	Sampling	Group	Sample size	Age (y)	Female (ratio), n (%)	Whether fall definition provided	Outcome (ascertainment method)	First fall	Participants (ratio) affected, n (%)	Follow-up
Wan et al (2026) [53]	China	Prospective cohort (2013‐2018)	Public database (CHARLS^a^)	Multi (28 provinces)	NR^b^	Sarcopenia	1087	71 (65‐76)^c^	745 (68.5)	No	Fall occurrence (self-reported)	No	246 (22.6)	6 y
Lin et al (2025) [54]	China	Prospective cohort (2015‐2018)	Public database (CHARLS)	Multi (28 provinces)	NR	—^d^	Rural: 2032; and urban: 3844	66 (63‐72)^c^	2961 (50.4)	No	Fall occurrence (self-reported)	No	1317 (22.4)	3 y
Takeshita et al (2025) [55]	Japan	Prospective cohort (2019‐2023)	EHR^e^ and telephone survey	Multi (4 hospitals)	Hospital based	Hospitalization history	706	Fall: 84.7 (6.2)^f^; and nonfall: 83.4 (6.7)^f^	425 (60.2)	Yes	Fall occurrence (self-reported)	No	114 (16.1)	3 mo
Park et al (2025) [56]	South Korea	Prospective cohort (2016‐2019)	Public database (KFACS^g^)	Multi (NR)	NR	Cognitive frailty	443	70-84^h^	363 (81.9)	No	Fall occurrence (self-reported)	No	NR	2 y
Liu et al (2023) [57]	United States	Retrospective case-control (NR)	EHR	Single	Hospital based	Abdominal indications	9029	66.6 (9.7)^f^	5728 (63.4)	No	Fall occurrence (EHR record)	No	3535 (39.2)	6.5 y
Silveira et al (2023) [58]	Brazil	Retrospective case-control (NR)	Clinical records and fitness center assessments	Multi (2 centers)	Clinic based	CVD^i^	72	Fall: 76.5 (65-92)^c^; and nonfall: 73 (60-86)^c^	29 (40.3)	Yes	Fall occurrence (self- or proxy-reported with staff monitoring)	No	24 (33.3)	1 y
Chen et al (2023a) [24]	China	Prospective cohort (2015‐2018)	Public database (CHARLS)	Multi (28 provinces)	NR	—	5818	67.7 (6.2)^f^	2982 (51.3)	No	Fall occurrence (self-reported)	No	1347 (23.2)	3 y
Chen et al (2023b) [28]	China	Prospective cohort (2011‐2018)	Public database (CHARLS)	Multi (28 provinces)	NR	—	1617	67.7 (6.2)^f^	697 (43.1)	No	Fall occurrence (self-reported)	No	235 (14.5)	7 y
Dormosh et al (2023) [59]	The Netherlands	Retrospective cohort (2018‐2019)	EHR	Multi (50 general practices)	Registry based	—	36,470	Fall: 76.6 (70.7- 83.3)^c^; and nonfall: 71.4 (68-77.1)^c^	19,398 (53.2)	No	Fall occurrence (EHR record, chart review)	No	4778 (13.1)	1 y
Ramsdale et al (2023) [60]	United States	Prospective cohort (NR)	Public database (GAP^j^70+ study)	Multi (NR)	Cluster randomized	Advanced cancer	522	≥70^h^	NR	No	Fall occurrence (self-reported)	No	89 (17)	3 mo
Ikeda et al (2022) [61]	Japan	Prospective cohort (2010‐2013)	Public database (JAGES^k^)	Multi (24 municipalities)	Registry based	—	61,883	Multiple falls: 75.4 (6.1)^f^; and no or annual fall: 72.8 (5.5)^f^	33,242 (53.7)	No	Multiple falls or not (self-reported)	No	3359 (5.4)	3 y
Mishra et al (2022) [30]	United States	Retrospective cohort (NR)	EHR	Single	Facility based	—	92	86.2 (6.4)^f^	57 (62)	No	Fall occurrence (staff reported)	No	31 (33.7)	6 mo
Kelly et al (2022) [62]	Sweden	Prospective cohort (NR)	Self-collected	Single	NR	—	1705	70	817 (47.9)	Yes	Fall occurrence (self-reported)	No	255 (15)	1 y
Dasgupta et al (2022) [29]	United States	Prospective cohort (2019)	Self-collected	Single	Convenience sampling	Hospitalization history	134	68.9 (8.1)^f^	54 (40.3)	Yes	Fall occurrence (self-reported)	No	14 (10.4)	3 mo
Tang and Romero-Ortuno (2022) [27]	Ireland	Prospective cohort (2009‐2016)	Public database (TILDA^l^)	Multi (NR)	NR	—	2900	≥65^h^	NR	Yes	Falls or syncope occurrence (self-reported)	No	1351 (46.6)	6 y
Cuaya-Simbro et al (2021) [63]	Mexico	Prospective cohort (NR)	Self-collected	Single	Clinic based	Women with osteoporosis	126	74.3 (6.3)^f^	126 (100)	No	Fall occurrence (self-reported)	No	NR	2.5 y
Omae et al (2021) [64]	Japan	Prospective cohort (2017‐2018)	Self-collected	Single	Community health checkup based	—	577	79 (77-83)^c^	304 (52.7)	No	Fall occurrence (self-reported)	No	201 (34.8)	1 y
Makino et al (2021) [65]	Japan	Prospective cohort (2011‐2016)	Self-collected	Single	NR	—	2520	71.1 (4.7)^f^	1303 (51.7)	Yes	Fall occurrence (self-reported)	No	415 (16.5)	4 y
Cuaya-Simbro et al (2020) [66]	Mexico	Prospective cohort (2019)	Self-collected	Multi (2 centers)	NR	Women	253	≥60^h^	253 (100)	No	Fall occurrence (self-reported)	No	First 6 mo: 38 (15%); Second 6 mo: 26 (10.3%)	1 y
Cella et al (2020) [67]	Italy	Prospective cohort (NR)	Self-collected	Single	Clinic based	—	96	77.2 (6.5)^f^	62 (64.6)	Yes	Fall occurrence (diary record)	No	32 (33.3)	1 y
Silva et al (2020) [68]	Portugal	Prospective cohort (NR)	Self-collected	Single	NR	—	281	75.1 (6.9)^f^	183 (65.1)	No	Fall occurrence (self-reported)	No	74 (26.3)	1 y
Ye et al (2020) [22]	United States	Retrospective cohort (2016‐2018)	EHR	Multi (69 facilities)	Registry based	—	265,225	≥65^h^	NR	Yes	Fall occurrence (EHR record, chart review)	No	4361 (1.6)	1 y
Kuspinar et al (2019) [69]	Canada	Prospective cohort (2002‐2014)	Administrative data	Multi (4 provinces)	Registry based	—	126,703	77 (14)^f^	82,357 (65)	No	Fall occurrence (self-reported)	No	NR	6 mo
Gillain et al (2019) [70]	Belgium	Prospective cohort (2014‐2017)	Self-collected	Single	Volunteer based	—	96	71.3 (5.4)^f^	48 (50)	Yes	Fall occurrence (diary record)	No	35 (36.5)	2 y
Howcroft et al (2018) [71]	Canada	Prospective cohort	Self-collected	Single	Convenience sampling	—	75	75.2 (6.6)^f^	44 (58.7)	Yes	Fall occurrence (self-reported)	No	28 (37.3)	6 mo
Deschamps et al (2016) [72]	France	Prospective cohort	Self-collected	Multi (NR)	NR	—	426	69.5 (2.6)^f^	265 (62.2)	Yes	Fall occurrence (self-reported, adjudicated by geriatric committee)	Yes	82 (19.2)	1 y
Marschollek et al (2011) [73]	Germany	Prospective cohort (2007‐2008)	Self-collected	Single	Hospital based	Hospitalization history	46	81.3^c^	37 (80.4)	Yes	Fall occurrence (self-reported)	No	19 (41.3)	1 y
Bath et al (2000) [74]	United Kingdom	Prospective cohort (1985‐1989)	Self-collected	Single	Random sampling	—	435	≥65^h^	NR	No	Fall occurrence (self-reported)	No	114 (26.2)	4 y

a CHARLS: China Health and Retirement Longitudinal Study.

b NR: not reported.

c Median (IQR).

d Not applicable.

e EHR: electronic health record.

f Mean (SD).

g KFACS: Korean Frailty and Aging Cohort Study.

h Age range.

i CVD: cardiovascular disease.

j GAP: geriatric assessment for patients.

k JAGES: Japan Gerontological Evaluation Study.

l TILDA: The Irish Longitudinal Study on Ageing.

Among them, 24 studies (85.7%) adopted a prospective design, while 4 (14.3%) were retrospective. Two (7.1%) were case-control studies [57,58], while others (92.9%) were cohort studies. Regarding data sources, 13 (46.4%) studies relied on investigator-collected datasets, 8 (28.6%) used public databases, 6 (21.4%) used electronic health records or clinical records, and 1 (3.6%) used administrative data. Thirteen (46.4%) studies were conducted in single-center settings, and 15 (53.6%) were multicenter studies.

With regard to the study population, 18 (64.3%) studies focused on general community-dwelling older adults, with fall incidence ranging from 1.6% to 46.6%. The remaining 10 studies targeted populations with specific conditions, including hospitalization history (n=3, 10.7%) [29,55,73], sarcopenia (n=1, 3.6%) [53], cognitive frailty (n=1, 3.6%) [56], abdominal indications (n=1, 3.6%) [57], cardiovascular disease (n=1, 3.6%) [58], advanced cancer (n=1, 3.6%) [60], women with osteoporosis (n=1, 3.6%) [63], and women in general (n=1, 3.6%) [66]. Sample sizes for model development ranged from 46 [73] to 265,225 [22]. The mean or median age of participants ranged from 66 [54] to 86.2 [30] years, and the proportion of female participants ranged from 40.3% [29,58] to 100% [63,66].

Twelve (42.9%) studies explicitly reported a precise fall definition, whereas the remaining 16 (57.1%) did not. Most studies (n=26, 92.9%) used fall occurrence as the outcome, and most outcomes were ascertained exclusively via self-report (n=20, 71.4%). Only one study (3.6%) [72] specifically predicted first-time falls.

### Model Development and Validation Characteristics

Table 2 summarizes the model development and validation characteristics of the included studies. Eleven (39.3%) studies reported internal validation split ratios, most commonly a 70% training and 30% test set (n=6, 54.5%). Eighteen (64.3%) studies applied cross-validation, with 10-fold cross-validation being the most frequently used approach (n=12, 66.7%). Among the 23 studies that reported feature selection methods, the most frequently applied was decision tree-based selection (n=4, 17.4%).

A total of 23 (82.1%) studies used ML techniques, while 5 (17.9%) applied dL. The most frequently used modeling approaches were decision tree (n=6, 21.4%) and RF (n=6, 21.4%).

Data modality included text, sensor, image, and multimodal sources. More than half of the studies (n=18, 64.3%) relied solely on conventional text data sources, including demographic variables, functional status, health conditions, psychological factors, medication use, behavioral factors, home environment, socioeconomic indicators, and prior fall history. The remaining studies incorporated sensor data (n=5, 17.9%), image data (n=1, 3.6%), or multimodal data (n=4, 14.3%).

**Table 2.** Summary of missing data handling methods, feature selection techniques, modeling approaches, data modalities, validation strategies, calibration assessments, and discrimination performance of included machine learning and deep learning models developed to predict future falls among community-dwelling older adults^a^.

Author (year)	Missing data	Data split ratio (train:test)	Cross-validation	Feature selection	Modeling	Data modality	Calibration, 95% CI or mean (SD)	AUC^b^ (95% CI)	Other discrimination metrics, 95% CI or mean (SD)
Wan et al (2026) [53]	Excluding, multiple imputation	70:30:00	10-fold	RF^c^	RF^d^	Text	NR^e^	0.97 (0.01)	Acc^f^: 92.26 (1.38); Sens^g^: 89.31 (2.85); Spec^h^: 95.19 (0.92); Precision: 94.86 (1.03); *F*_1_: 91.98 (1.55)
Lin et al (2025) [54]	Excluding, algorithm imputation	70:30:00	10-fold	LASSO^i^	RF^d^	Text	Rural: Brier score: 0.150; urban: Brier score: 0.159	Rural: 0.732 (0.685‐0.782); urban: 0.724 (0.687‐0.757)	Rural: Acc: 0.667; Sens: 0.669; Spec: 0.667; *F*_1_: 0.456; and urban: Acc: 0.754; Sens: 0.519; Spec: 0.825; *F*_1_: 0.495
Takeshita et al (2025) [55]	KNN^j^, mode	70:30:00	5-fold	Information gain	Extra trees^d^	Text	NR	0.73^d^	Acc: 0.69; Sens: 0.68; Spec: 0.69; PPV^k^: 0.29; NPV^l^: 0.92; *F*_1_: 0.41
Park et al (2025) [56]	NR	NR	NR	RFE^m^	LR^n^	Text	NR	0.956 (0.953‐0.958)	Acc: 0.916 (0.912‐0.920); Sens: 0.937 (0.933‐0.941); Spec: 0.910 (0.906‐0.914)
Liu et al (2023) [57]	Excluding	NR	NR	NR	U-Neta	Image	NR	0.657	NR
Silveira et al (2023) [58]	Excluding	NR	5-fold	RFE-RF	RF^c^	Text	NR	NR	Acc: 0.76; Sens: 0.68
Chen et al (2023^o^) [24]	MissForest algorithm for imputation	70:30:00	10-fold	LASSO	LR^d^	Text	Brier score: 0.158	0.739 (0.690‐0.777)	Acc: 0.671; Sens: 0.707; Spec: 0.659
Chen et al (2023^d^) [28]	Multiple imputation	70:30:00	10-fold	NR	RF^d^	Text	Brier score: 0.170 (0.163‐0.176)	0.731 (0.721‐0.735)	Acc: 0.830 (0.824‐0.831); Sens: 0.545 (0.544‐0.546); PPV: 0.667 (0.664‐0.668)
Dormosh et al (2023) [59]	Excluding	NR	10-fold	LASSO	LR	Text	Calibration plots	0.718 (0.708‐0.727)	NR
Ramsdale et al (2023) [60]	Excluding, Median imputation	80:20:00	NR	Ensemble feature selection	MLP^d^^,^^o^^,^^p^	Text	NR	0.75	NR
Ikeda et al (2022) [61]	Excluding, RF imputation	NR	Nested 10-fold	RF-based Boruta algorithm	XGBoost^d^^,^^q^	Text	NR	0.88±0.02	Acc: 0.88 (0.02); *F*_1_: 0.89 (0.02)
Mishra et al (2022) [30]	Excluding	NR	5-fold	NR	SVM^d^^,^^r^	Text	Brier score: 0.15	0.80 (0.76‐0.85)	Acc: 0.75 (0.72‐0.79); Sens: 0.82 (0.74‐0.89); Spec: 0.72 (0.67‐0.76); *F*_1_: 0.76 (0.72‐0.79)
Kelly et al (2022) [62]	NR	75:25:00	10-fold	Automated machine learning with Bayesian optimization	SGD^d^^,^^s^	Sensor	NR	NR	Sens: 0.61 (0.49‐0.71); Spec: 0.66 (0.61‐0.71)
Dasgupta et al (2022) [29]	No missing	NR	NR	Wald test	HCRNN^o^^,^^t^	Sensor	NR	0.99 (0.98‐1)	NR
Tangand Romero-Ortuno (2022) [27]	Excluding	NR	NR	NR	RF	Text	NR	NR	Precision: 0.63; Sens: 0.56; *F*_1_: 0.59
Cuaya-Simbro et al (2021) [63]	NR	NR	10-fold	FSMC^u^	RF^d^	Sensor	NR	NR	Sens: >0.71; Spec: >0.18; PPV: >0.74; NPV: >0.66
Omae et al (2021) [64]	Multiple imputation	80:20:00	NR	RF	Decision tree	Text	NR	0.818	Acc: 0.836; Sens: 0.884; Spec: 0.766; PPV: 0.847; NPV: 0.818
Makino et al (2021) [65]	Excluding	NR	10-fold	Decision tree	Decision tree^d^	Text	NR	0.70 (0.68‐0.72)	Acc: 0.65 (0.64‐0.67); Sens: 0.62 (0.60‐0.64); Spec: 0.69 (0.67‐0.71); PPV: 0.66 (0.64‐0.69); NPV: 0.64 (0.62‐0.66)
Cuaya-Simbro et al (2020) [66]	NR	NR	10-fold	FSMC	DBN^d^^,^^o^^,^^v^	Sensor	NR	NR	First 6 months: Acc: 0.800; Sens: 0.737; Precision: 0.700; F1: 0.718; and second 6 months: Acc: 0.882; Sens: 0.626; Precision: 0.831; *F*_1_: 0.714
Cella et al (2020) [67]	NR	NR	Nested 5-fold	NR	LASSO	Text, sensor	NR	0.81 (0.72‐0.90)	Precision: 0.69; Sens: 0.78; Spec: 0.74
Silva et al (2020) [68]	NR	67:33:00	NR	PCA^w^ and variance threshold method	Decision tree^d^	Text, sensor	NR	0.505 (0.043)	Acc: 0.370 (0.098); PPV: 0.262 (0.032); Sens: 0.786 (0.222); Spec: 0.225 (0.199); *F*_1_: 0.382 (0.060)
Ye et al (2020) [22]	NR	67:33:00	10-fold	Univariate logistic regression	XGBoost^d^	Text	Isotonic regression	0.807	PPV: 0.110
Kuspinar et al (2019) [69]	NR	70:30:00	NR	Decision tree	Decision tree	Text	NR	Internal: 0.60; external: 0.55, 0.58, 0.58	NR
Gillain et al (2019) [70]	NR	NR	10-fold	Decision tree	Decision tree	Text, sensor	NR	0.84	Acc: 0.84; Sens: 0.80; Spec: 0.87; PPV: 0.78; NPV: 0.88
Howcroft et al (2018) [71]	NR	75:25:00	10-fold	Relief-F, fast correlation–based filter	SVM^d^	Sensor	NR	NR	Acc: 0.56; Sens: 0.59; Spec: 0.69; PPV: 0.56; NPV: 0.73; *F*_1_: 0.56
Deschamps et al (2016) [72]	Algorithm	80:20:00	NR	Decision tree	Decision tree	Text	NR	0.72	Sens: 0.82; Spec: 0.61
Marschollek et al (2011) [73]	NR	NR	10×10-fold	Wrapper subset evaluator	LR^d^	Text, sensor	Brier score: 0.20	0.74	Acc: 0.72; Sens: 0.68; Spec: 0.74; PPV: 0.65; NPV: 0.77
Bath et al (2000) [74]	NR	NR	LOOCV^x^	GANN^y^	GANN^o^	Text	NR	NR	Acc: 0.76; Sens: 0.31; Spec: 0.92; PPV: 0.57; NPV: 0.79

a For studies discussing multiple models, only the best-performing model and its corresponding performance metrics are presented. The AUC was reported based on validation data when available. Only Kuspinar et al (2019) [69] reported external validation. Some studies reported AUC with 95% CI and others as mean (SD); these formats represent different types of variability and cannot be interconverted. Values are therefore presented as originally reported. Accuracy and *F*_1_-score are threshold- and prevalence-dependent measures. When reported without accompanying discrimination (eg, AUC) and calibration metrics, their interpretability and comparability across studies are limited.

b AUC: area under the curve.

c RF: random forest.

d Studies reporting multiple models trained on the same dataset; the listed modeling method and performance correspond to the best-performing model.

e NR: not reported.

f Acc: accuracy.

g Sens: sensitivity.

h Spec: specificity.

i LASSO: Least Absolute Shrinkage and Selection Operator.

j KNN: K‑nearest neighbor.

k PPV: positive predictive value.

l NPV: negative predictive value.

m RFE: recursive feature elimination.

n LR: logistic regression.

o Deep learning.

p MLP: multilayer perceptron.

q XGBoost: Extreme Gradient Boosting.

r SVM: support vector machine.

s SGD: stochastic gradient descent.

t HCRNN: hybrid-convolutional recurrent neural network.

u FSMC: feature selection for minority class.

v DBN: dynamic Bayesian network.

w PCA: principal component analysis.

x LOOCV: Leave-one-out cross-validation.

y GANN: genetic algorithm neural network.

Table S1 in Multimedia Appendix 1 presents the full details of the final predictors in each model. Table 3 concludes predictors used in at least three models, with the 5 most frequent being age, sex, fall history, depression, and basic activities of daily living (BADL-ADL), appearing in 15, 13, 12, 11, and 9 models, respectively. Other frequently included predictors were walking speed, chair stand test results, cognition, hypertension, fracture history, vision, and the use of cardiovascular drugs.

**Table 3.** Summary of the frequently selected predictors in included machine learning–based and deep learning–based fall risk prediction models among community-dwelling older adults.

Category and predictors^a^	Participants, n
Demographic factors	
Age	15
Sex	13
Marital status	3
Functions, gait, and balance	
BADL^b^ or ADL^c^^,^^d^	9
Walking/gait speed	6
Chair stands test/rising from a chair	6
TUG^e^	5
IADL^f^^,^^g^	5
Grip or hand strength	4
Gait-related parameters (sensor)	4
Balance-related parameters (sensor)	4
Balance/Berg balance scale	3
Assistance with walking	3
Comorbidity and health status	
Cognition	6
Hypertension	6
Fracture or injury history	6
Vision or vision impairment	6
BMI	5
Diabetes	5
Arthritis or osteoarthritis	5
Incontinence	5
Memory	5
Pain	5
Cancer	4
Stroke	4
Hearing or hearing impairment	4
CVD^h^	3
Parkinson disease	3
Osteoporosis	3
Self-rated health	3
Teeth or teeth number	3
Psychological factor	
Depression	11
Lifestyles	
Sleeping duration or time asleep	5
Smoking	4
Drinking or alcohol abuse	3
Medication-related factors	
Cardiovascular drugs	6
Polypharmacy	5
Psychiatric and neurological drugs	5
Number of taken medications	4
Fall-related factors	
Fall history	12
Fear of falling	3

a Predictors were extracted from the final predictive model reported in each included longitudinal study. Full details of the final predictors in each model are presented in Table S1 in Multimedia Appendix 1.

b BADL: basic activity of daily living.

c ADL: activity of daily living.

d ADL and BADL were grouped together as they both reflect core self-care functions in the included studies.

e TUG: Timed Up and Go.

f IADL: instrumental activity of daily living.

g IADL was presented separately due to its distinct role in assessing instrumental daily living skills.

h CVD: cardiovascular disease.

### Results of Individual Studies

Table 2 also summarizes the performance of the included prediction models. In terms of discrimination, 20 models (71.4%) reported AUC or c-index values, which ranged from 0.505 [68] to 0.99 [15]. Calibration was assessed in 7 (25%) studies [22,24,28,30,54,59,73], with 5 (17.9%) reporting Brier scores ranging from 0.15 [30,54] to 0.20 [73]. Only one model underwent external validation [69], while 2 models did not report either internal or external validation [27,57].

Figure S1 in Multimedia Appendix 1 shows that among studies reporting both the sample and AUC, over half of the studies (n=14/21, 66.7%) used classic ML with text data across all sample sizes, yielding median AUCs of approximately 0.73 to 0.81. DL evidence was limited, appearing mainly in nontext modalities and in studies with moderate sample sizes, with variable median AUCs (0.66‐0.99) based on few studies.

### Risk of Bias and Applicability

Table 4 presents the risk of bias and applicability assessments for the included prediction models. All models were assessed as having a high risk of bias, suggesting methodological limitations during model development and/or validation. In the participants domain, 5 models were thought to be at high risk of bias due to retrospective data sources [22,30,57-59]. In the predictors domain, 11 models were rated as high risk, primarily due to inadequate predictor definitions, measurement methods, or failure to clearly report which variables were ultimately included in the final model [22,24,27-29,53,59,60,62,63,68].

**Table 4.** Risk of bias and applicability assessment of included machine learning and deep learning models for predicting future falls in community-dwelling older adults, evaluated using the PROBAST^a^.

Author (year)	Study type	ROB^b^				Applicability			Overall	
		Participants	Predictors	Outcome	Analysis	Participants	Predictors	Outcome	ROB	Applicability
Wan et al (2026) [53]	B^c^	+^d^	-^e^	-	-	-	+	+	-	-
Lin et al (2025) [54]	B	+	+	-	-	+	+	+	-	+
Takeshita et al (2025) [55]	B	+	+	-	-	-	+	+	-	-
Park et al (2025) [56]	B	+	+	-	-	-	+	+	-	-
Liu et al (2023) [57]	A^f^	-	+	-	-	-	+	+	-	-
Silveira et al (2023) [58]	B	-	+	-	-	-	+	+	-	-
Chen et al (2023a) [24]	B	+	-	-	-	+	-	+	-	-
Chen et al (2023b) [28]	B	+	-	-	-	+	-	+	-	-
Dormosh et al (2023) [59]	B	-	-	-	-	+	-	+	-	-
Ramsdale et al (2023) [60]	B	+	-	-	-	-	-	+	-	-
Ikeda et al (2022) [61]	B	+	+	-	-	+	+	-	-	-
Mishra et al (2022) [30]	B	-	+	-	-	+	+	+	-	+
Kelly et al (2022) [62]	B	+	-	-	-	+	-	+	-	-
Dasgupta et al (2022) [29]	B	+	-	-	-	-	-	+	-	-
Tang and Romero-Ortuno (2022) [27]	A	+	-	-	-	+	-	+	-	-
Cuaya-Simbro et al (2021) [63]	B	+	-	-	-	-	-	+	-	-
Omae et al (2021) [64]	B	+	+	-	-	+	+	+	-	+
Makino et al (2021) [65]	B	+	+	-	-	+	+	+	-	+
Cuaya-Simbro et al (2020) [66]	B	+	+	-	-	-	+	+	-	-
Cella et al (2020) [67]	B	+	+	-	-	+	+	+	-	+
Silva et al (2020) [68]	B	+	-	-	-	+	-	+	-	-
Ye et al (2020) [22]	B	-	-	?	-	+	-	+	-	-
Kuspinar et al (2019) [69]	C^g^	+	+	-	-	+	+	+	-	+
Gillain et al (2019) [70]	B	+^d^	+	-^e^	-	+	+	+	-	+
Howcroft et al (2018) [71]	B	+	+	?^h^	-	+	+	+	-	+
Deschamps et al (2016) [72]	B	+	+	-	-	+	+	+	-	+
Marschollek et al (2011) [73]	B	+	+	-	-	-	+	+	-	-
Bath et al (2000) [74]	B	+	+	-	-	+	+	+	-	+

a PROBAST: Prediction model Risk of Bias Assessment Tool.

b ROB: risk of bias.

c B: ”development and internal or cross-validation in the same publication.”

d +: low ROB or low concern regarding applicability.

e -: high ROB or high concern regarding application.

f A: “development only.”

g C: “development, internal validation, and external validation in the same publication.”

h ?: unclear ROB or unclear concern regarding applicability.

In the outcome domain, all models were assessed as having either a high or unclear risk of bias, with all studies lacking blind assessments between predictors and the outcome. Sixteen (57.1%) studies only reported fall occurrence as the outcome without providing a precise definition of a fall [24,28,30,53,54,56,57,59-61,63,64,66,68,69,74], and 20 (71.4%) studies relied on self-reported or caregiver-reported outcome records, introducing potential bias [24,27-29,53-56,60-69,72,74].

Regarding the analysis domain, all models were classified as high risk of bias. Twelve (42.9%) studies had fewer than 10 events per variable, indicating insufficient sample size for reliable model estimation [28-30,53,55,58,66,67,70-72,74]. Six (21.4%) studies excluded cases with missing data [27,30,57-59,65], and 11 (39.3%) studies did not describe their methods for handling missing data [22,56,62,63,66-71,74]. Seven (25%) studies did not report the AUC or c-index [27,58,62,63,66,71,74], and 9 (32.1%) studies reported insufficient details regarding other model discrimination metrics [22,29,57-61,69,72]. Calibration assessments were absent in 21 (75%) studies [27,29,53,55-58,60-72,74], and 6 (21.4%) studies had potential risks of overfitting, underfitting, or optimism in model performance [27,53,56,57,68,69]. In terms of validation, only one model underwent external validation [69], while 2 (7.1%) models lacked both internal and external validation [27,57]. The remaining models reported internal validation only, of which 13 (26.4%) relied on random data splitting rather than more robust approaches such as cross-validation or bootstrapping.

Concerning applicability, 18 (64.3%) studies were considered to be of high concern, while 10 (35.7%) were rated as low concern. In the participants domain, 10 (35.7%) studies exhibited high applicability concerns due to the restriction of the study sample to specific subgroups, such as older adults with particular diseases, or single-sex populations [29,53,55-58,60,63,66,73]. In the predictors domain, 10 (35.7%) studies were rated as high concern due to unclear definitions, final inclusion, or measurement of predictors [22,24,27-29,59,60,62,63,68]. In the outcome domain, one study demonstrated high applicability concern due to the construction of a binary outcome variable based on multiple falls versus a combination of single fall and nonfall cases, thereby limiting real-world interpretability [61]. On the basis of the recurrent sources of bias identified by PROBAST in the included studies, we summarize key methodological considerations as a more detailed checklist to inform future research in ML-based fall risk prediction (Table S5 in Multimedia Appendix 1).

### Synthesis of Results

**Figure 3.**
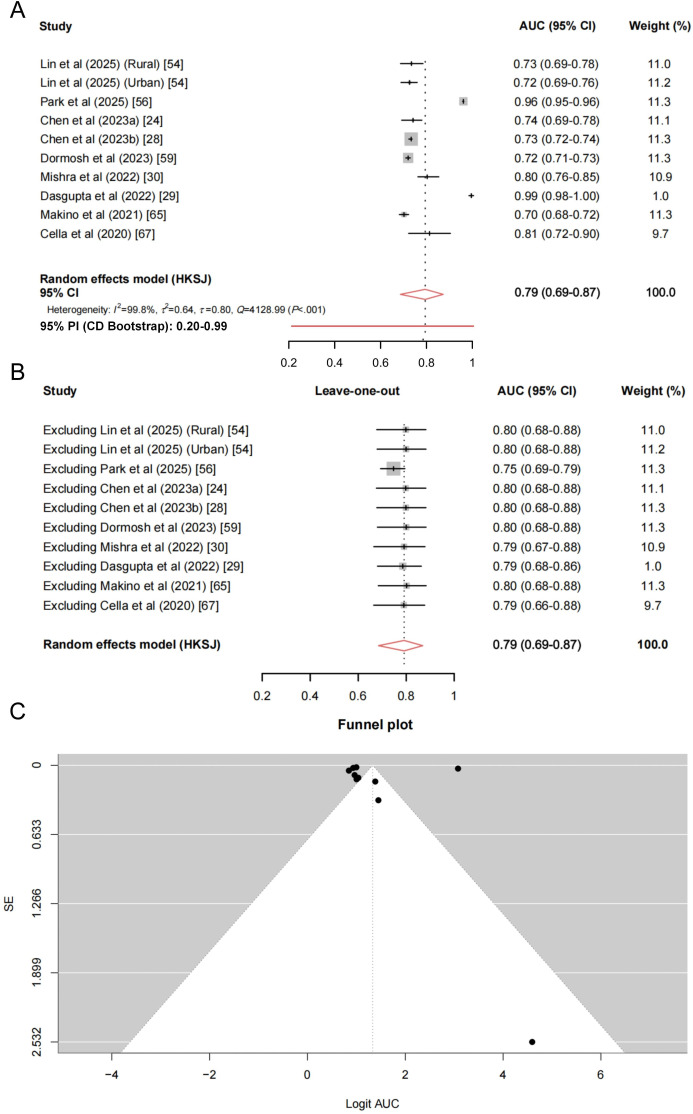
Random-effects meta-analysis of model discrimination performance using the best-performing model from each cohort. (A) Forest plot presenting individual model areas under the curve (AUCs) with corresponding 95% CIs and the pooled AUC estimated using the Hartung-Knapp-Sidik-Jonkman (HKSJ) method (back-transformed from the logit scale). Between-study heterogeneity is quantified using *I*^2^, *τ*^2^, *τ,* and *Q* statistic. 95% Prediction interval (PI; confidence-distribution [CD] bootstrap) is also reported in the pooled summary to illustrate the expected range of model performance in future populations. (B) Leave-one-out sensitivity analysis, in which the pooled AUC is recalculated after sequential exclusion of each model to assess the robustness of the overall estimate. (C) Funnel plot of logit-transformed AUC values to evaluate potential small-study effects [24,28-30,54,56,59,65,67].

The data extraction sheets underlying the meta-analysis are provided in Table S6 in Multimedia Appendix 1. Ten models evaluated using internal validation were included in the primary quantitative synthesis, with the AUC used as the effect size to assess the discriminative performance of these ML-based fall risk prediction models. As only one model underwent external validation and did not provide adequate information to compute SE, it was excluded from the quantitative synthesis and instead summarized qualitatively. The pooled discriminative performance was good, with a back-transformed pooled AUC of 0.79 (95% CI 0.69‐0.87) under the HKSJ random-effects model. Substantial between-study heterogeneity was present (*τ*^2^=0.64; *τ*=0.80; *I*²=99.8%; *Q*=4128.99; *P*<.001; Figure 3A [24,28-30,54,56,59,65,67]).

To further characterize the expected dispersion of model performance in new populations, the main forest plot presents the 95% PI estimated using the confidence-distribution bootstrap method, which ranged from 0.20 to 0.99 (Figure 3A). PIs estimated using alternative methods were similarly wide (HTS: 0.28‐0.98; HTS-HK: 0.40‐0.96; and HTS-SJ: 0.40‐0.96), leading to the same qualitative interpretation of substantial uncertainty in real-world generalizability.

Within-study averaging meta-analysis showed that the pooled discriminative performance remained comparable to the primary model-level analysis using best-performing models, with a back-transformed pooled AUC of 0.77 (95% CI 0.65‐0.86) under the HKSJ random-effects model. Between-study heterogeneity was similarly high (*τ*^2^=0.74; *τ*=0.86; *I*^2^=99.9%; *Q*=6353.03; *P*<.001), indicating substantial true variability across study cohorts (Figure S2A in Multimedia Appendix 1). The supplementary forest plot presents the 95% PI estimated using the confidence-distribution bootstrap method, which ranged from 0.29 to 0.97 (Figure S2A in Multimedia Appendix 1). PIs estimated using alternative methods were similarly wide (HTS: 0.35‐0.96; HTS-HK: 0.35‐0.96; HTS-SJ: 0.35‐0.96), supporting the same qualitative interpretation of substantial uncertainty in expected performance across new populations.

### Additional Analyses

Leave-one-out sensitivity analyses for the primary analysis showed that the pooled AUC was stable (range: 0.75‐0.80), suggesting that no single study disproportionately influenced the overall estimate (Figure 3B). As several included models were derived from the same large population-based database (CHARLS), additional sensitivity analyses were conducted to evaluate the potential impact of correlated cohorts on the pooled estimates. Sequential analyses retaining only one CHARLS-derived model at a time yielded consistent results, with pooled AUC estimates of 0.82 (95% CI 0.65‐0.92) when retaining the rural model developed by Lin et al [54], 0.82 (95% CI 0.65‐0.92) when retaining the urban model developed by Lin et al [54], 0.82 (95% CI 0.66‐0.92) when retaining the model developed by Chen et al [24], and 0.82 (95% CI 0.65‐0.92) when retaining the model developed by Chen et al [28]. An additional sensitivity analysis excluding all 4 CHARLS-derived models also produced a comparable pooled estimate (AUC 0.84, 95% CI 0.63‐0.94), despite the reduced number of models (n=6). These findings indicate that the overall pooled discrimination estimate was robust and not materially driven by models derived from the same database.

Funnel plot inspection for the primary analysis did not reveal pronounced asymmetry, and the Egger regression test was nonsignificant (*t*_8_=0.16; *P*=.88). However, these findings should be interpreted cautiously because asymmetry tests have limited power and are difficult to interpret when the number of studies is small (Figure 3C).

Similarly, for the within-study averaging meta-analysis, leave-one-out sensitivity analyses showed minimal influence of any single study cohort, with pooled AUC estimates remaining stable across iterations (range: 0.72‐0.78; Figure S2B in Multimedia Appendix 1). Funnel plot inspection and Egger regression test likewise did not suggest strong evidence of small-study effects (*t*_8_=1.33; *P*=.22), although interpretation remains limited by the small number of samples and high heterogeneity (Figure S2C in Multimedia Appendix 1).

Subgroup analyses were conducted to explore potential sources of heterogeneity. For each subgroup, pooled AUCs with corresponding 95% CIs, heterogeneity statistics (*I*^2^, *τ*^2^, *τ*, and *Q* statistic) are presented in Figures S3-S6 in Multimedia Appendix 1, with the 95% PI estimated using the confidence-distribution bootstrap method displayed. Prediction time window (≤1 y vs >1 y) did not significantly moderate discriminative performance (*P*=.95) and explained none of the observed heterogeneity. Similarly, no significant difference was observed between text-based models and sensor-based or multimodal models (*P*=.58); estimates for the latter subgroup were imprecise due to the small number of included studies.

Sample size (≤500 vs >500 participants) significantly moderated model discrimination. Meta-regression demonstrated a statistically significant subgroup effect (*P*=.02), accounting for approximately 42% of between-study heterogeneity, with higher AUCs observed in models developed from smaller samples, consistent with potential optimistic performance estimates. Population type (general vs. specific conditions) also emerged as a significant moderator (*P*<.001), explaining 87% of the observed heterogeneity, with higher discrimination in models developed for specific populations compared with general community-dwelling older adults; however, this finding should be interpreted cautiously given the limited number of studies in the specific population subgroup.

## Discussion

### Summary of Findings

Falls are a leading cause of injury, disability, and loss of independence among older adults [75-79]. Unlike institutionalized older adults under continuous professional care, community-dwelling older adults often lack intensive monitoring, making early identification of high-risk community-dwelling individuals critical for timely prevention [8]. Overall, our findings indicate that current ML-DL models show promising discriminative ability for predicting future falls. Nonetheless, their readiness for real-world implementation is limited by a lack of external validation, insufficient calibration assessment, and consistently high risk of bias. In this review, we systematically synthesized evidence from 28 studies evaluating ML-DL–based prediction models for future falls in community-dwelling older adults. These models incorporated a range of predictors, most commonly age, sex, fall history, depression, and BADL-ADL, reflecting the multifactorial nature of fall risk. Although the models generally showed acceptable discrimination, their performance varied considerably, and the available evidence was based largely on internal rather than external validation. Methodological quality assessment indicated that all included models were at high risk of bias, and meta-analyses revealed substantial between-study heterogeneity. Taken together, these findings highlight both the potential and current limitations of ML-DL–based approaches and provide an evidence base to inform the development of more robust and clinically applicable fall risk prediction tools for proactive prevention in community settings.

Table S4 in Multimedia Appendix 1 contrasts the scope and methodological focus of key prior reviews with our systematic review. Prior reviews have emphasized fall risk management but mainly focused on cross-sectional risk classification, real-time fall detection systems, or experimental sensor-based technologies, rather than predictive models for primary prevention [18,35,37,80]. While detection systems are valuable for acute response, their capacity to inform proactive, upstream prevention strategies is limited [81,82].

Early risk identification through cost-effective prediction models is essential for implementing targeted prevention, addressing modifiable risk factors, and ultimately reducing fall incidence [83]. A recent review [36] primarily focusing on conventional statistical models, such as logistic regression and Cox regression, reported moderate to good discrimination (AUC 0.65‐0.88) but highlighted their limited ability to capture complex interactions and integrate diverse data sources. ML approaches, in contrast, can accommodate high-dimensional data and capture nonlinear dependencies [31], making them better suited for multifactorial fall risk modeling. More recently, Xie et al [35] reviewed ML models for in-hospital fall prediction, offering insights into short-term risk management but providing limited guidance for broader, longer-term prevention in the community.

To our knowledge, no systematic review has comprehensively evaluated ML-based models for predicting future falls in older adults in community settings, where early identification and long-term prevention are most needed [18,35-37,80]. Importantly, longitudinal community-based fall risk prediction differs fundamentally from in-hospital screening or real-time detection tasks in several methodological respects. Such models rely on baseline predictors measured prior to outcome occurrence, require explicit definition of prediction horizons, and must appropriately account for censoring and loss to follow-up [84,85]. Moreover, because predicted risks are intended to guide preventive decision-making, adequate calibration across time horizons is essential, rather than discrimination alone [85,86]. Failure to recognize these distinctions risks conflating analytically and clinically distinct tasks. By restricting inclusion to longitudinal, community-based studies, this review addresses this gap and clarifies the unique methodological challenges and opportunities of ML-based approaches for proactive fall prevention.

The included studies showed substantial variation in geographic distribution and sample characteristics. Most were conducted in East Asia, North America, or Europe, with limited representation from low- and middle-income regions. This imbalance may limit generalizability, as fall risk profiles may differ substantially in underrepresented populations due to disparities in health care access, living environments, and socioeconomic factors [87]. Moreover, all included studies focused on single-ethnicity populations, further limiting our understanding of how these models perform across diverse racial or cultural groups. Sample sizes also varied considerably. Models developed from small datasets are particularly prone to overfitting [88], especially when lacking validation. Additionally, nearly half of the studies were single center, which may limit model robustness, while multicenter designs are better positioned to enhance generalizability. Importantly, although all included participants were classified as community-dwelling, recruitment sources were not uniform across studies. Some cohorts were sampled from the general community, whereas others were recruited through clinically enriched pathways. This distinction is important because community-dwelling status refers to living arrangement rather than recruitment context. For example, 1 of the 2 studies in the specific population subgroup recruited older adults being discharged home from the emergency department, representing community-living individuals with recent acute-care contact rather than a general community sample [29]. The other focused on community-dwelling older adults with cognitive frailty, a clinically defined high-risk subgroup within the broader community [56]. Consistent with this distinction, our subgroup analysis showed significantly higher discrimination in models developed for specific populations than in models developed for general community-dwelling older adults, with population subgroup explaining a large proportion of the observed heterogeneity. However, this finding should be interpreted cautiously because the specific population subgroup included only 2 studies, and these represented different forms of clinical enrichment. Nonetheless, it suggests that apparently stronger model performance may partly reflect narrower case mix and more concentrated risk profiles, rather than better transportability to the broader community-dwelling older population.

Nearly all studies predicted fall occurrence without distinguishing between first-time and recurrent events, with only one model targeting first falls [72]. This represents a critical gap, as the risk factors and prevention strategies for first falls differ from those for recurrent falls [89]. First-time falls often mark the transition into frailty and can precipitate fear of falling, reduced activity, and long-term disability. In contrast, recurrent fallers may already be known to their family or caregivers and are more likely to receive fall-related interventions [90,91]. Developing separate prediction models for these 2 groups could enhance clinical utility and enable more targeted preventive interventions. Accordingly, the first fall prediction should be treated as a distinct modeling objective with tailored populations, predictor sets, prediction horizons, and decision thresholds; related practical recommendations are provided in Box S1 in Multimedia Appendix 1.

Frequently selected predictors included age, sex, fall history, depression, and BADL. Age was the most common predictor, which is plausible given that with aging, progressive declines in neuromuscular control, sensory function, and balance increase the likelihood of instability [92]. Age is also associated with multimorbidity, polypharmacy, and sarcopenia, which further exacerbate vulnerability to falls and fall-related injuries [93,94]. Sex was another recurrent predictor, with women generally facing a higher risk, partly due to osteoporosis and postmenopausal bone loss [95], as well as differences in muscle mass and hormonal profiles [96,97].

Fall history consistently served as a strong predictor because intrinsic or extrinsic causes of previous falls often remain unsolved and increase recurrence risk. Repeated falls may trigger fear of falling and activity avoidance, which in turn contribute to physical deconditioning and further functional decline [98]. Depression may raise fall risk both directly, through psychomotor slowing and fatigue, and indirectly, through physical inactivity and antidepressant use [99,100]. ADL-BADL reflects basic functional capacity; difficulties with tasks such as bathing or dressing often indicate underlying mobility or balance deficits and signal elevated fall risk [101,102]. Broader risk factors were also included, including motor functions, health status, lifestyle behaviors, and medication use. Prior research highlighted the importance of both motor and nonmotor factors in fall risk [103].

Our review included 23 ML-based and 5 DL-based models, reflecting the current predominance of ML in fall prediction research. Rather than demonstrating a clear superiority of one approach over the other, our findings suggest that ML and DL are each better suited to different data modalities. ML models were primarily developed using structured, text-based predictors, such as demographics, health status, medication use, lifestyle behaviors, and fall history, which are relatively inexpensive to collect and thus facilitate cost-effective fall prediction in community settings.

DL models were predominantly applied to sensor or image data, which provide high-dimensional, continuous signals capable of capturing subtle patterns not easily represented in structured variables. Although DL shows considerable potential in leveraging such complex data types, its application remains constrained by challenges in data acquisition, large sample size requirements, high computational resources, and limited interpretability [104,105]. Among the DL studies, the input data were primarily high-dimensional signals, including raw or minimally processed multivariate inertial sensor streams (eg, accelerometer or gyroscope gait time series) and imaging-derived matrices. Accordingly, the reported architectures spanned convolutional neural networks (CNNs) for local pattern extraction, recurrent units (eg, long short-term memory or gated recurrent unit) for sequential dependency modeling, and hybrid CNN-RNN designs (eg, hybrid-convolutional recurrent neural network) for end-to-end sequence learning; for imaging tasks, encoder-decoder CNNs such as U-Net support multiscale feature extraction with spatially explicit outputs. Figure S1 in Multimedia Appendix 1 suggests that DL is selectively applied to high-dimensional, nontext modalities, whereas classic ML predominates for structured data across all sample sizes. Although DL shows high median AUCs in some strata, these results are based on few studies, highlighting uncertainty in generalizability and reinforcing the need for cautious interpretation and external validation.

In our review, 9 models used sensor-based gait and balance parameters, including multimodal assessments. These models have the advantage of capturing dynamic abnormalities in movement patterns that are not easily detected through traditional clinical assessments. However, predictor definitions, measurements, and reports varied across studies, undermining the reproducibility and comparability of these models. Together, these findings underscore the need for more comprehensive and holistic modeling approaches to achieve effective prediction.

Across the 28 included studies, reported AUC values were generally acceptable but varied widely, indicating considerable variability in predictive performance across different models and study settings. However, only one model underwent external validation, but it could not be included in the quantitative synthesis due to insufficient information to derive SE. This limited use of external validation substantially constrains the ability to judge model performance in real-world settings and increases the likelihood that reported results are optimistic. All remaining models relied exclusively on internal validation approaches, such as cross-validation or random data splitting. As internal validation is known to yield optimistically biased performance estimates, these results primarily reflect within-sample discrimination and provide limited evidence regarding model performance in independent, real-world populations [106].

The meta-analysis of models with internal validation indicated apparently acceptable discrimination. However, the pooled AUC should not be interpreted as a robust estimate of real-world predictive performance. Rather, it represents a summary of reported discrimination from studies that were uniformly judged to be at high risk of bias and were based almost entirely on internal validation. In this context, an AUC of 0.79 is better understood as indicating that these models can separate higher-risk from lower-risk individuals under study-specific conditions, but that the magnitude of this performance is likely to be inflated relative to what would be expected in independent community populations. In the absence of external validation, the current evidence base primarily reflects methodological performance under controlled conditions and remains insufficient to support direct clinical implementation of ML-based fall risk prediction models.

Interpretation of this pooled estimate is further limited by extremely high between-study heterogeneity, suggesting substantial variation in study design, populations, prediction horizons, data modalities, and model inputs rather than random error alone. Accordingly, the pooled AUC should be viewed as an average across highly diverse, high-risk-of-bias studies rather than as a stable benchmark for model performance. Nevertheless, pooling remained informative by quantifying the central tendency of reported discrimination and, critically, by enabling estimation of PIs that explicitly convey the expected dispersion of model performance in new populations. Importantly, PIs provide information beyond pooled estimates by reflecting the range of model performance that may be expected in new settings, rather than only the average discrimination across studies [48,107]. This is particularly relevant for ML-based prediction models, where performance is often context-dependent and influenced by differences in populations, data sources, feature definitions, and model development strategies. In such settings, pooled AUC values alone may give an overly optimistic or simplified impression of performance, whereas PIs explicitly capture the uncertainty and variability that are likely to be encountered in real-world applications. Therefore, reporting PIs alongside pooled estimates is essential for a more realistic assessment of generalizability and for informing decisions about model implementation in diverse community settings. Consistent pooled estimates obtained from leave-one-out sensitivity analyses and within-study averaged AUCs further suggest that no single study disproportionately influenced the overall results, but this stability should not be mistaken for low bias or strong transportability.

To systematically explore sources of heterogeneity, we conducted prespecified subgroup analyses and mixed-effects meta-regression. These analyses demonstrated that sample size and population subgroup significantly moderated discriminative performance, jointly explaining a substantial proportion of heterogeneity, whereas prediction time window and data modality did not materially affect pooled AUCs. Notably, PIs were wide across all subgroups, regardless of the estimation method, highlighting considerable uncertainty in real-world generalizability even when average discrimination appears acceptable.

Importantly, the absence of a statistically significant moderating effect of prediction time window should not be interpreted as clinical equivalence across different horizons. Models predicting short-term fall risk are conceptually aligned with transitional care and near-term preventive interventions, whereas models estimating multiyear fall risk may be more suitable for long-term prevention planning or population-level surveillance. These application scenarios differ fundamentally in terms of intervention timing, decision thresholds, update frequency, and calibration requirements. The wide variation in prediction horizons therefore reflects heterogeneity in clinical intent rather than merely methodological noise, underscoring that models developed for one time horizon should not be directly extrapolated to another without revalidation [84,85].

Beyond discrimination, additional methodological limitations further restrict clinical interpretability. All included models were rated as having a high risk of bias, reflecting common shortcomings, such as insufficient sample sizes, exclusion of missing data without clear justification, preprocessing pipelines, limited reporting of feature definitions, unclear definition of retained predictors, and reliance on self- or proxy-reported fall events. Calibration, which assesses agreement between predicted risks and observed outcomes, was rarely reported: only 5 studies provided Brier scores, of which only one reported a corresponding 95% CI, and only a single study presented a calibration plot, precluding quantitative synthesis of calibration performance. This lack of calibration assessment further limits the clinical interpretability of these models, as accurate risk estimation is essential for decision-making, threshold selection, and implementation in real-world care pathways. Consequently, most models were evaluated primarily using uncalibrated AUCs, limiting their utility for threshold-based risk stratification, shared decision-making, and intervention planning. For this reason, the pooled AUC should not be taken as evidence that these models are sufficiently reliable for deployment, but rather as a provisional summary of reported discrimination within a methodologically weak evidence base. Collectively, these methodological shortcomings compromise internal validity, hinder reproducibility, and substantially constrain the potential for real-world translation.

Unlike prior reviews [18,35-37,80], we comprehensively evaluated ML-based risk prediction models developed specifically to predict future falls among community-dwelling older adults from a preventive perspective. By including only longitudinal studies, the findings are directly relevant to real-world fall prediction rather than post hoc risk classification. This review focuses on longitudinal ML-DL models for predicting future falls in community-dwelling older adults, thereby extending beyond prior reviews centered on real-time detection, in-hospital fall prediction, or conventional statistical models. By integrating PROBAST appraisal with HKSJ meta-analysis, PIs, and modality-specific mapping of algorithm families, the review provides a structured and methodologically grounded synthesis to interpret both average discrimination and between-study variability. These findings help contextualize reported model performance and highlight key considerations for evaluating generalizability and uncertainty across different settings.

### Limitations

Some limitations should be acknowledged. First, all included studies were at high risk of bias, and the predominance of models with internal validation limits confidence in generalizability. Only one model underwent external validation, and this study could not be included in the quantitative synthesis due to insufficient reporting. As a result, the pooled AUC should be interpreted as a summary of reported discrimination from a methodologically weak evidence base, rather than as a robust estimate of transportable performance in real-world settings. Second, substantial heterogeneity remained despite prespecified subgroup analyses and meta-regression, reflecting fundamental differences in study populations, designs, and modeling approaches. Some potentially relevant factors, such as algorithm type, validation strategy, outcome definition, and geographic region, could not be examined quantitatively due to data limitations. This heterogeneity further limits the extent to which any single pooled estimate can be viewed as a stable benchmark for expected model performance. In addition, the category of community dwelling was not equivalent to uniform population-based recruitment across studies. Some included cohorts were recruited through emergency care or clinically defined high-risk pathways rather than from the general community, and our subgroup analysis suggested that models developed in specific populations showed higher discrimination than those developed in general community-dwelling samples. However, because the specific population subgroup comprised only 2 studies with different types of clinical enrichment, this finding should be interpreted cautiously and cannot establish a stable or broadly generalizable subgroup effect. Third, calibration was infrequently reported, limiting assessment of clinical usability, as discrimination alone is insufficient for risk stratification and decision support. In addition, inconsistent reporting of performance metrics, limited transparency in feature selection and data preprocessing, and restricted data availability further constrain reproducibility and real-world applicability. Accordingly, even models with apparently acceptable discrimination may still provide poorly calibrated or overly optimistic risk estimates when applied outside their development settings. Fourth, several models were derived from the same database, which may introduce partial correlation between effect estimates. Although some models were developed in distinct subpopulations or analytic cohorts, participant overlap cannot be fully excluded. However, sensitivity analyses showed that pooled AUC estimates were similar when retaining only one CHARLS-derived model or excluding all such models, suggesting minimal impact on the overall conclusions. Finally, the restriction to English-language studies and the exclusion of preprints may have introduced language and publication bias, which should be considered when interpreting the findings.

From a translation perspective, the wide PIs indicate that models with apparently acceptable average discrimination may perform substantially differently when applied to new community settings. This is particularly important given that the available pooled estimate is derived largely from internally validated, high-risk-of-bias studies and may therefore overstate real-world predictive performance. Accordingly, these tools should be considered as decision-support aids rather than stand-alone screening instruments, and implementation should be accompanied by local recalibration and context-specific threshold selection [85].

Future research should prioritize prospective, multicenter studies with rigorous external validation and standardized outcome definitions. Transparent reporting of both discrimination and calibration, as well as predictor handling and data processing, is essential; at a minimum, AUC should be reported alongside calibration measures [104,108]. In addition, impact studies, interventional trials, and silent trials are needed to determine whether these models improve patient outcomes and resource use. Public availability of feature dictionaries, preprocessing code, and calibration information will further support reproducibility and safe real-world translation. Until such evidence is available, pooled discrimination estimates should be viewed primarily as signals of potential utility rather than evidence of implementation readiness.

### Conclusions

This systematic review and meta-analysis demonstrates growing interest in the use of ML-DL to predict future falls among older adults, but the current evidence should be interpreted cautiously. Although pooled discrimination appeared promising, substantial between-study heterogeneity, wide PIs, uniformly high risk of bias, limited external validation, and sparse calibration assessment indicate that the reported AUC is better understood as a summary of study-specific performance than as a robust estimate of transportable real-world accuracy. This caution is further warranted because “community dwelling” did not always correspond to recruitment from the general community, and some models were developed in clinically enriched community-living subgroups.

By focusing on longitudinal ML-DL models for predicting future falls in community-dwelling older adults, this review extends prior work centered on real-time detection, in-hospital prediction, or conventional statistical models. Through the integration of PROBAST appraisal, HKSJ meta-analysis, PIs, and modality-specific synthesis, it provides a decision-relevant framework for interpreting both discrimination and uncertainty across settings. At present, the evidence supports the potential utility of ML-DL models as decision-support tools for proactive fall prevention, but external validation, calibration assessment, local recalibration, and context-specific threshold selection remain essential before implementation.

## Supplementary material

10.2196/84844 Multimedia Appendix 1 Additional supporting materials for the systematic review and meta-analysis, including database search strategies, detailed model characteristics, and supplementary analyses.

10.2196/84844 Checklist 1 PRISMA 2020 checklist.

## Abbreviations

ADL: activity of daily living
AUC: area under the curve
BADL: basic activity of daily living
CHARLS: China Health and Retirement Longitudinal Study
CNN: convolutional neural network
DL: deep learning
GAIDeT: Generative Artificial Intelligence Delegation Taxonomy
Gen-AI: generative artificial intelligence
HKSJ: Hartung-Knapp-Sidik-Jonkman
HTS: Higgins-Thompson-Spiegelhalter
MeSH: Medical Subject Headings
ML: machine learning
PI: prediction interval
PRISMA: Preferred Reporting Items for Systematic Reviews and Meta-Analyses
PRISMA-DTA: Preferred Reporting Items for Systematic Reviews and Meta-Analyses Statement for Diagnostic Test Accuracy
PRISMA-S: Preferred Reporting Items for Systematic Reviews and Meta-Analyses literature search extension
PROBAST: Prediction Model Risk of Bias Assessment Tool
RF: random forest
