# Supplementary material for: Machine Learning and Deep Learning Models for Predicting Future Falls in Community-Dwelling Older Adults: Systematic Review and Meta-Analysis of Longitudinal Evidence
Source: J Med Internet Res. 2026 May 14;28:e84844. doi: 10.2196/84844 (PMC13175450; doi:10.2196/84844)
Supplement: Multimedia Appendix 1 [file jmir-v28-e84844-s001.docx]

Supplementary Materials

*Search Strategies*

*PubMed Database*

#1 elder*[Title/Abstract] OR old[Title/Abstract] OR senior*[Title/Abstract] OR geriatric*[Title/Abstract] OR age[Title/Abstract] OR aged[Title/Abstract] OR aging[Title/Abstract]

#2 aged[MeSH Terms]

#3 Artificial intelligence[Title/Abstract] OR Machine learning[Title/Abstract] OR Smart home system*[Title/Abstract] OR Home smart renovation[Title/Abstract] OR Wearable device*[Title/Abstract] OR Digital health[Title/Abstract] OR Telemonitor*[Title/Abstract] OR AI[Title/Abstract] OR smart sensor*[Title/Abstract]

#4 (artificial intelligence[MeSH Terms]) OR (machine learning[MeSH Terms])

#5 fall*[Title/Abstract] OR slip*[Title/Abstract] OR trip*[Title/Abstract]

#6 accidental fall[MeSH Terms]

#7 (#1 OR #2) AND (#3 OR #4) AND (#5 OR #6)

*Embase Database*

#1 'elder*':ab,ti OR 'old':ab,ti OR 'senior*':ab,ti OR 'geriatric*':ab,ti OR 'age':ab,ti OR 'aged':ab,ti OR 'aging':ab,ti OR 'aged'/exp

#2 'artificial intelligence':ab,ti OR 'machine learning':ab,ti OR 'smart home system*':ab,ti OR 'home smart renovation':ab,ti OR 'wearable device*':ab,ti OR 'digital health':ab,ti OR 'telemonitor*':ab,ti OR 'ai':ab,ti OR 'smart sensor*':ab,ti OR 'artificial intelligence'/exp OR 'machine learning'/exp

#3 'fall*':ab,ti OR 'slip*':ab,ti OR 'trip*':ab,ti OR 'accidental fall'/exp

#4 #1 AND #2 AND #3

*Web of Science Core Collection*

((TS=(elder* OR old OR senior* OR geriatric* OR age OR aged OR aging)) AND TS=(Artificial intelligence OR Machine learning OR Smart home system* OR Home smart renovation OR Wearable device* OR Digital health OR Telemonitor* OR AI OR smart sensor*)) AND TS=(fall* OR slip* OR trip*)

*CINAHL Database*

#1 TI (elder* OR old OR senior* OR geriatric* OR age OR aged OR aging) OR AB (elder* OR old OR senior* OR geriatric* OR age OR aged OR aging) OR MH aged

#2 TI (Artificial intelligence OR Machine learning OR Smart home system* OR Home smart renovation OR Wearable device* OR Digital health OR Telemonitor* OR AI OR smart sensor*) OR AB (Artificial intelligence OR Machine learning OR Smart home system* OR Home smart renovation OR Wearable device* OR Digital health OR Telemonitor* OR AI OR smart sensor*) OR MH (artificial intelligence OR machine learning)

#3 TI (fall* OR slip* OR trip*) OR AB (fall* OR slip* OR trip*) OR MH (accidental fall)

#4 #1 AND #2 AND #3

*Cochrane Database*

#1 (elder* OR old OR senior* OR geriatric* OR age OR aged OR aging):ti,ab,kw

#2 MeSH descriptor: [Aged] explode all trees

#3 (Artificial intelligence OR Machine learning OR Smart home system* OR Home smart renovation OR Wearable device* OR Digital health OR Telemonitor* OR AI OR smart sensor*):ti,ab,kw

#4 MeSH descriptor: [Artificial Intelligence] explode all trees

#5 MeSH descriptor: [Machine Learning] explode all trees

#6 (fall* OR slip* OR trip*):ti,ab,kw

#7 MeSH descriptor: [Accidental Falls] explode all trees

#8 (#1 OR #2) AND (#3 OR #4 OR #5) AND (#6 OR #7)

*IEEE Database*

"All Metadata":elder* OR "All Metadata":old OR "All Metadata":senior* OR "All Metadata":geriatric* OR "All Metadata":age OR "All Metadata":aged OR "All Metadata":aging) AND ("All Metadata":Artificial intelligence OR "All Metadata":Machine learning OR "All Metadata":Smart home system* OR "All Metadata":Home smart renovation OR "All Metadata":Wearable device* OR "All Metadata":Digital health OR "All Metadata":Telemonitor* OR "All Metadata":AI OR "All Metadata":smart sensor*) AND ("All Metadata":fall* OR "All Metadata":slip* OR "All Metadata":trip*)

*Table S1.* Summary of basic information of the included prediction models.

| **Author (y)** | **Modality** | **Final variables used in predictions** |
| --- | --- | --- |
| Wan et al (2026) [1] | Text | **1) Demographics:** Marital status, Education;  **2) Health status:** Self-rated health, Visual impairment, Hearing impairment, 12 comorbidities (eg, Hypertension, Hyperlipidemia, Memory-related diseases, Arthritis or rheumatism), Blood pressure and blood glucose control, Hip fracture, Glaucoma, Dentition status, Pain, Memory;  **3) Psychological factors:** Depression (CESD-10 scale score);  **4) Functions:** ADL, IADL, Assistive device usage, Mobility;  **5) Lifestyles:** Physical activities, Social participation, Recreational social participation, Smoking, Nap duration;  **6) Home environment:** Indoor cleanliness, Indoor temperature;  **7) Socioeconomic variables and family characteristics:** Personal assets, Personal income, Debt status, Number of siblings, Number of living children, Financial support from children, Financial support for parents, Whether the father is alive, Whether the father is currently working. |
| Lin et al (2025) [2] | Text | **Rural:**  **1) Demographics:** Age, Sex, Child number, Marital status, Live alone;  **2) Health status:** Diabetes;  **3) Psychological factors:** Depression (CESD-10 scale score);  **4) Functions:** ADL, IADL, Chair stands test;  **5) Medication:** Psychiatric and Neurological: Memory medicine;  **6) Home environment:** House y, Internet;  **7) Fall experience:** Fall history.  **Urban:**  **1) Demographics:** Age, Sex, Occupation;  **2) Health status:** Memory, Hypertension, Liver disease, Heart diseases, Kidney disease, Psychiatric problems, Arthritis, Pain, Physical disabilities, Experience of hip fracture, Stroke, Dental care in the past y, Have physical examination in the past 2 ys, Waist, BMI;  **3) Psychological factors:** Life satisfaction, Children relation satisfaction, Depression (CESD-10 scale score);  **4) Functions:** ADL, Lung function, Hand strength, Balance, Chair stands test, Walking speed;  **5) Medications:**  Psychiatric and Neurological: Memory medicine, Psychiatric medicine;  Cardiovascular: Dyslipidemia medicine, Heart medicine, Stroke medicine;  Other: Digestive medicine;  **6) Lifestyles:** Sleeping duration;  **7) Home environment:** Residence used for business, Kitchen, Bathroom facilities, Have coal gas or natural gas supply, Telephone, Internet, Tidiness;  **8) Fall experience:** Fall history. |
| Takeshita et al (2025) [3] | Text | **1) Demographics:** Age, Family composition;  **2) Health status:** Obesity, Leg circumference, Weight, Diarrhea, Sleep disorder, Delirium, Clinical Frailty Scale, Clinical Frailty Scale change, Urinary incontinence, Newly acquired incontinence;  **3) Psychological factors:** Depression (GDS-15);  **4) Functions:** IADL, BADL (Barthel index);  **5) Lifestyles:** Smoking;  **6) Medication:** Polypharmacy;  **7) Home environment:** Prehospital residence;  **8) Fall experience:** Fall history. |
| Park et al (2025) [4] | Text | **1) Health status:**Number of Fried physical frailty phenotypes;  **2) Functions:** PF-Mobility scores, Simple Five item Scoring Scale for Sarcopenia (SARC-F) scores (consisting of five components: strength, assistance with walking, rising from a chair, climbing stairs, and experiencing falls);  **3) Psychosocial factor:** Short Geriatric Depression Scale (SGDS-K) scores. |
| Liu et al (2023) [5] | Image | **1) CT biomarkers:** Low L1 muscle HU, High L1TAT area, Low L1 BMD. |
| Silveira et al (2023) [6] | Text | **1) Functions:** VO2_max_; Dual-task in seconds (8-foot up-and-go test with verbal fluency test); Berg balance scale score;  **2) Medications:**  Cardiovascular: β-blockers, Anticoagulants, Diuretics. |
| Chen et al (2023a) [7] | Text | **1) Demographics:** Sex;  **2) Health status:** Vision, Diabetes, Liver disease, Memory related disease, Brain disabilities, Vision disabilities, Physical disabilities, Experience of hip fracture, Dental care;  **3) Psychosocial factor:** Depression (CESD-10 scale score);  **4) Functions:** BADL, IADL, Walking speed, Hand strength;  **5) Lifestyles:** Smoking, Drinking, Sleeping duration;  **6) Medications:**  Psychiatric and Neurological: Memory medicine;  Cardiovascular: Antihypertensives, Heart medicine;  Analgesics and Anti-inflammatories: Arthritis medicine;  Other: Lung medicine, Kidney medicine;  **7) Home environment:** Structure of building; Handicapped facilities; Kitchen; Flushed toilets; Cooking fuel; Internet; House tidiness; House temperature;  **8) Socioeconomic variables:** Relationship with children, Health satisfaction;  **9) Biomarkers:** White Blood Cell, Blood Urea Nitrogen, Glucose;  **10) Vital:** SBP;  **11) Fall experience:** Fall history. |
| Chen et al (2023b) [8] | Text | **1) Demographics:** Age, Sex, Residence, Marital status, Occupation, Education, Geographical location, Household registration;  **2) Health status:** Vision, Hearing, Comorbidity, Teeth, Body pain, Self-rated memory, Major misfortune injury experience, Self-reported health status, Disability, Cognition, BMI;  **3) Psychosocial factors:** Depression (CESD-10 scale score);  **4) Functions:** ADL, Grip strength, Balance, Walking speed, Abdominal obesity, Chair stands test, Lung function;  **5) Medication:** Polypharmacy;  **6) Lifestyles:** Smoking, Drinking, Sleeping duration, Social activities;  **7) Socioeconomic factors:** Income, Life satisfaction, Medical service, Medical insurance. |
| Dormosh et al (2023) [9] | Text | **Clinical variables:**  **1) Demographics:** Age, Sex;  **2) Health status:** Parkinson’s disease, Memory and concentration problems, Vertigo and dizziness, Urinary incontinence, Stroke, Fatigue and weakness, Osteoporosis, Other mental disorders, Osteoarthritis, Epilepsy, Vision, Orthostatic hypotension, Cancer, Hearing, Neurological cephalalgia, Cardiac arrhythmia, Diabetes, Fractures, Chronic skin problems, Allergy, Back or neck disorders, Hypertension, Previous injury, Vitamin deficiencies;  **3) Psychosocial factors:** Depression;  **4) Lifestyles:** Alcohol abuse;  **5) Medications:**  Psychiatric and Neurological: Antiparkinson drugs, Opioids, Antiepileptics, SSRIs, Non selective monoamine reuptake inhibitors;  Cardiovascular: Antiarrhythmics, Statins, ARBs;  Antidiabetic: Insulins;  Other: Urinary incontinence drugs, Proton pomp inhibitors.  **Topics extracted from clinical notes:** Residential care, Cognition, Cardiovascular risk management among others, Urine test, Preventive and diagnostic care mainly diabetes, Bladder cancer, Blood pressure measurement, Hypertension, Erectile dysfunction, Pre-travel health advice and vaccination, New patient registration or unregistering. |
| Ramsdale et al (2023) [10] | Text | **1) Health status:** Impaired cognition, Lung cancer, OARS comorbidity score, 6-month weight loss;  **2) Functions:** ADL, SPPB, KPS;  **3) Fall experience:** Number of prior falls. |
| Ikeda et al (2022) [11] | Text | **1) Demographics:** Age;  **2) Health status:** Self-rated health, Choking experience, Dry mouth, Arthrosis, Incontinence, Number of remaining teeth;  **3) Psychosocial factors:** Depression (Japanese Geriatric Depression Scale), Sense of coherence scale score;  **4) Functions:** Ability to stand up from chairs without using one’s hands, Difficulties in eating hard foods, Ability to climb stairs without a handrail;  **5) Fall experience:** Number of falls, Fear of falling. |
| Mishra et al(2022) [12] | Text | **1) Demographics:** Age, Sex;  **2) Health status:** Cognition (MMSE), SF12-Physical Component Summary;  **3) Psychosocial factors:** Depression (GDS), SF12-Mental Component Summary;  **4) Functions:** ADL, IADL, FAP scores, Gait speed;  **5) Fall experience:** Fall history within 6 months. |
| Kelly et al (2022) [13] | Sensor | **1) Gait:** Step-based features (Vertical/horizontal acceleration, peak timing, variance, entropy), Frequency-based features (FFT phase at 1.9 Hz & 5.5 Hz, CWT coefficients). |
| Dasgupta et al (2022) [14] | Sensor | **1) Function:** TUG;  **2) Balance parameters:** accelerometer parameters. |
| Tang et al(2022) [15] | Text | **1) Demographics:** Age, Sex;  **2) Health status:** Osteoarthritis, Osteoporosis, Hypertension, Urine incontinence, Abnormal heart rhythm, Angina, COPD;  **3) Functions:** Grip weakness, Unsteady getting up from chair;  **4) Medications:**  Psychiatric and Neurological: Z-drugs, Antidepressants;  Cardiovascular: Antihypertensives;  Other: Anticholinergics, Polypharmacy;  **5) Fall experience:** Fall in last y, Fear of falling, Frequent fainter when young, History of blackout/faint. |
| Cuaya-Simbro et al(2021) [16] | Sensor | **1) Balance parameters:** COP Displacement, COP Velocity, Sway Area, Romberg coefficient. |
| Omae et al (2021) [17] | Text | **1) Demographics:** Age, Sex;  **2) Health status:** Visual impairment, Dementia, Diabetes, Hypertension, Stroke, BMI;  **3) Psychosocial factors:** Depression;  **4) Functions:** Overactive bladder status, Gait speed. |
| Makino et al (2021) [18] | Text | **1) Demographics:** Age;  **2) Health status:** Lower limb pain;  **3) Functions:** TUG;  **4) Medications:** Polypharmacy;  **5) Fall experience:** Fall history, Fear of falling. |
| Cuaya-Simbro et al(2020) [19] | Sensor | **1) Gait:** Spatial (Step length, stride length, base of support), Temporal (Cadence, Ambulation time, Step/cycle time, Step/cycle time differentials). |
| Cella et al (2020) [20] | Text, Sensor | **1) Demographics:** Age, Sex;  **2) Functions:** TUG, Tinetti POMA, SPPB, Gait Speed;  **3) Medications:** Number of medications taken daily;  **4) Fall experience:** Fall history;  **5) Balance parameters (20 Robotic parameters):** eg, postural control, stability limits, compensatory trunk movements. |
| Silva et al(2020) [21] | Text, Sensor | **1) Demographics:** Age, Sex;  **2) Functions:** TUG, 30s Sit-to-Stand repetition, Time to stands;  **3) Medications:** Number of medications, Polypharmacy;  **4) Fall experience:** Fall history;  **5) Balance parameter:** Postural sway. |
| Ye et al (2020) [22] | Text | **1) Demographics:** Age, Sex;  **2) Health status:**  Cognitive disorders: Alzheimer’s disease, dementia, amnesia;  Neurological: Parkinson’s disease, epilepsy, cerebral infarction;  Musculoskeletal: Osteoporosis, muscle disorders, abrasion of knee;  Cardiovascular: Heart failure, orthostatic hypotension;  Other: Diabetes complications, chronic kidney disease;  Number of diagnoses;  **3) Medications:**  Psychiatric and Neurological: SSRIs, SNRIs, TCAs, benzodiazepines  Cardiovascular: β-blockers, Loop diuretics, ACE inhibitors  Analgesics and Anti-inflammatories: NSAIDs, Opioid agonists  Other: Antiepileptics, Cholinergic muscarinic antagonists  Number of medications;  **4) Clinical Utilization:** Historical medical costs;  **5) Fall Experience:** Fall history. |
| Kuspinar et al (2019) [23] | Text | **1) Demographics:** Age, Sex.  **2) Health Status:** Cognition (Cognitive Performance Scale), Pain Scale, Parkinsonism, Unstable health patterns, Bladder incontinence;  **3) Functions:** ADL, managing medication, mobility in bed, worsening of ADL status, Primary mode of locomotion indoors (ambulatory/non-ambulatory), Unsteady gait. |
| Gillain et al (2019) [24] | Text, Sensor | **1) Demographics:** Sex;  **2) Function:** Stiffness (UPDRS);  **3) Gait:** Symmetry DTW cost, FW stride length, CW mean MTC, DTW CV MTC cost, FW variance MTC, FW mean MTC, DTW delta1 MTC. |
| Howcroft et al (2018) [25] | Sensor | **1) Gait:** Plantar-pressure parameters, accelerometer parameters |
| Deschamps et al (2016) [26] | Text | **1) Health status:** Nutrition (Mini Nutritional Assessment score), BMI, Lean body mass;  **2) Functions:** Ankle hypoesthesia, Visual acuity, Presbycusis (hearing loss), COP metrics during quiet standing (eyes open/closed), Limited knee range of motion, Foot pathology (eg, hallux valgus). |
| Marschollek et al (2011) [27] | Text, Sensor | **1) Demographics:** Age, Sex;  **2) Health status:** BMI;  **3) Functions:** BADL (Barthel Index), TUG;  **4) Fall Risk Assessment:** STRATIFY score. |
| Bath et al (2000) [28] | Text | **1) Health Status:**  Arthritis/rheumatism, Self-rated health, Shortness of breath when walking uphill, Headaches, Left-handed/ambidextrous;  **2) Psychosocial factors:** Depression (SAD depression score);  **3) Functions:** Pushing/dragging heavy loads frequency, Total flexibility score, Walking aid use;  **4) Medication:** Prescribed drug count;  **5) Lifestyle:** Time asleep;  **6) Socioeconomic variables:** Pet ownership, Religious/club attendance, Financial difficulty. |

*Abbreviation: ADL, Activities of Daily Living; IADL, Instrumental Activity of Daily Living; BADL, Basic Activity of Daily Living; PF, Physical Frailty; NSAID, Non-steroidal Anti-inflammatory Drugs; HU, Hounsfield unit; TAT, Total adipose tissue; BMD, Bone mineral density; CESD Scale, Center for Epidemiologic Studies Depression Scale; SBP, Systolic Blood Pressure; SSRI, Selective Serotonin Reuptake Inhibitors; ARBs, Angiotensin II Receptor Blockers; OARS Comorbidity Score, Older Americans Resources and Services Comorbidity Score; SPPB, Short Physical Performance Battery; KPS, Karnofsky Performance Status; MMSE, Mini-Mental State Examination; SF12, Short Form 12; FAP, Functional Ambulatory Profile; GDS, Geriatric Depression Scale; FFT, Fast Fourier Transform; CWT, Continuous Wavelet Transform; TUG, Timed Up and Go; COPD, Chronic Obstructive Pulmonary Disease; COP, Center of Pressure; POMA, Performance-Oriented Mobility Assessment; SPPB, Short Physical Performance Battery; TCA, Tricyclic antidepressants; DTW, Dual-task Walking; FW, Fast Walking; CW, Comfortable Walking; MTC, Minimum Toe Clearance; CV, Coefficient of Variation; [UPDRS](https://www.sciencedirect.com/topics/nursing-and-health-professions/unified-parkinsons-disease-rating-scale), Unified [Parkinson's Disease](https://www.sciencedirect.com/topics/pharmacology-toxicology-and-pharmaceutical-science/parkinsons-disease) Rating Scale; BMI, Body Mass Index.*

**
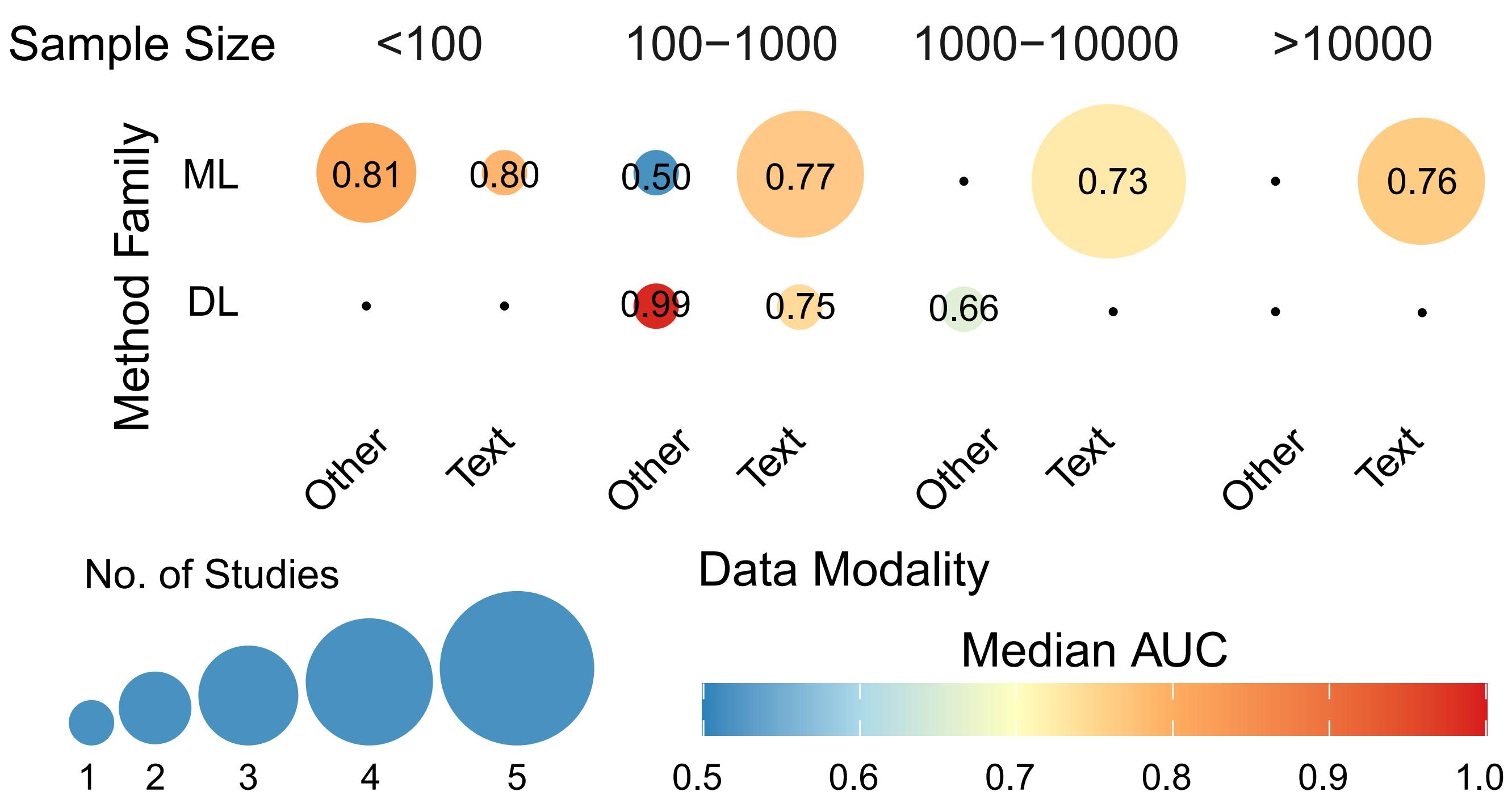
**

*Figure S1.* Evidence map of model discrimination (AUC) by method family, data modality, and sample size. The grid summarizes included studies of future fall-risk prediction in community-dwelling older adults, stratified by sample-size bins (<100, 100-1000, 1000-10,000, >10,000), method family (ML vs DL), and data modality (text vs other modalities, as defined in Table 2). Within each cell, the circle color represents the median area under the receiver operating characteristic curve (AUC) across studies in that cell (scale shown), and the circle size is proportional to the number of studies contributing to that cell (legend shown). Numeric labels indicate the median AUC where available; dots denote strata with no eligible studies (or insufficient AUC data) for summary display. This evidence map provides a visual overview of where DL has been applied (predominantly in non-text, higher-dimensional modalities) and where classic ML dominates (primarily structured/tabular predictors), facilitating interpretation of whether DL is likely justified given modality and available sample size.

*Table S2.* Methodological checklist for future ML-based fall-risk prediction studies.

| Domain | Key methodological recommendation |
| --- | --- |
| Participants | Clearly define the target population and recruitment design (prospective vs retrospective), and fully report sample characteristics, including sample size, age distribution (mean/median and range), sex composition, and follow-up duration. |
|  | Describe inclusion and exclusion criteria in sufficient detail, particularly age thresholds, sex requirements, and any restriction to specific clinical or functional subgroups. |
|  | Ensure an adequate sample size relative to model complexity. |
|  | Use multicenter data when feasible to enhance representativeness. |
| Predictors | Prespecify candidate predictors based on clinical relevance or prior evidence. |
|  | Clearly define each predictor, including measurement method, timing, and data source. |
|  | Describe feature processing steps (eg, normalization, encoding, aggregation). |
|  | Explicitly report which predictors were retained in the final model. |
| Outcome | Provide a clear, standardized definition of “fall”, explicitly stating the outcome construct and coding scheme. |
|  | Fully report outcome information (eg, the number of outcome events, the number of fallers and non-fallers, event rate). |
|  | Report outcome ascertainment method (eg, self-report, caregiver report, objective assessment). |
|  | Describe whether outcome assessment was blinded to predictor information, and discuss potential bias if blinding was not feasible. |
|  | Distinguish first-time falls from recurrent falls, or provide a clear justification for combining them into a single outcome. |
|  | Report follow-up duration in detail and align it explicitly with the stated prediction horizon, ensuring consistency between outcome timing and model purpose. |
| Analysis | Explicitly report the amount of missing data and clearly justify the handling strategy, including whether complete-case analysis or imputation was used; when applicable, describe the imputation method (eg, multiple imputation) and variables included. |
|  | Explicitly report events-per-variable (EPV) for model development; an EPV ≥10 is suggested as a minimum benchmark to reduce overfitting risk. |
|  | Prefer robust internal validation approaches, such as bootstrapping or nested cross-validation, over single train-test splits to obtain less optimistic performance estimates. |
|  | Conduct and report external validation using independent datasets to assess model transportability; clearly distinguish external from internal validation results. |
|  | Report discrimination performance using threshold-independent metrics, such as AUC or c-index; Also report complementary measures (eg, sensitivity, specificity, precision) with explicit thresholds. |
|  | Report calibration performance, including calibration plots and/or quantitative metrics (eg, Brier score, calibration slope and intercept), to assess agreement between predicted risks and observed outcomes. |
|  | Avoid selective reporting of only best-performing models or tuning results; transparently report model selection procedures and, where applicable, performance of candidate models. |
|  | Assess and discuss risks of overfitting, underfitting, and optimism, including how modeling choices, sample size, and validation strategy may influence reported performance estimates. |
| Applicability | Ensure that the study population reflects the intended target population for model deployment to support clinical transferability. |
|  | Use predictors that are realistically available and measurable in the intended care setting; avoid reliance on features that require specialized equipment or retrospective data sources unless justified. |
|  | Outcome constructions should be interpretable and actionable in practice. |

*Table S3.* Model-level discrimination estimates (AUC with 95% confidence intervals) of all eligible prediction models for future falls in community-dwelling older adults, with identification of best-performing models included in the primary meta-analysis and prespecified subgroup classification.

| Study (y) | Modelling | AUC | 95% CI (Lower) | 95% CI (Upper) | Included in primary meta-analysis (Best-performing per cohort) | Prediction time window | Sample size | Data modality | Population subgroup |
| --- | --- | --- | --- | --- | --- | --- | --- | --- | --- |
| Lin et al (2025) (Rural) [2] | RF | 0.732 | 0.685 | 0.782 | Yes | ＞1 y | ＞500 | Text | General |
|  | LR | 0.712 | 0.662 | 0.764 |  |  |  |  |  |
|  | SVM | 0.667 | 0.609 | 0.718 |  |  |  |  |  |
|  | XGBoost | 0.713 | 0.664 | 0.765 |  |  |  |  |  |
|  | LightGBM | 0.699 | 0.650 | 0.752 |  |  |  |  |  |
| Lin et al (2025) (Urban) [2] | RF | 0.724 | 0.687 | 0.757 | Yes | ＞1 y | ＞500 | Text | General |
|  | LR | 0.727 | 0.690 | 0.758 |  |  |  |  |  |
|  | SVM | 0.671 | 0.634 | 0.705 |  |  |  |  |  |
|  | XGBoost | 0.706 | 0.669 | 0.738 |  |  |  |  |  |
|  | LightGBM | 0.696 | 0.658 | 0.730 |  |  |  |  |  |
| Park et al (2025) [4] | LR | 0.956 | 0.953 | 0.958 | Yes | ＞1 y | ≤500 | Text | Specific |
| Chen et al (2023a) [7] | LR | 0.739 | 0.690 | 0.777 | Yes | ＞1 y | ＞500 | Text | General |
|  | SVM | 0.696 | 0.643 | 0.753 |  |  |  |  |  |
|  | RF | 0.711 | 0.660 | 0.749 |  |  |  |  |  |
|  | AdaBoost | 0.734 | 0.678 | 0.775 |  |  |  |  |  |
|  | LightGBM | 0.706 | 0.664 | 0.74 |  |  |  |  |  |
| Chen et al (2023b) [8] | RF | 0.731 | 0.721 | 0.735 | Yes | ＞1 y | ＞500 | Text | General |
|  | SVM | 0.604 | 0.599 | 0.605 |  |  |  |  |  |
|  | Decision tree | 0.68 | 0.677 | 0.681 |  |  |  |  |  |
|  | LR | 0.726 | 0.722 | 0.729 |  |  |  |  |  |
| Dormosh et al (2023) [9] | LR | 0.718 | 0.708 | 0.727 | Yes | ≤1 y | ＞500 | Text | General |
| Mishra et al (2022) [12] | SVM | 0.80 | 0.76 | 0.85 | Yes | ≤1 y | ≤500 | Text | General |
|  | LR | 0.77 | 0.71 | 0.84 |  |  |  |  |  |
|  | Decision tree | 0.63 | 0.56 | 0.71 |  |  |  |  |  |
|  | KNN | 0.78 | 0.73 | 0.82 |  |  |  |  |  |
|  | RF | 0.78 | 0.74 | 0.83 |  |  |  |  |  |
| Dasgupta et al (2022) [14] | HCRNN | 0.99 | 0.98 | 1.00 | Yes | ≤1 y | ≤500 | Sensor or  Multimodal | Specific |
| Makino et al (2021) [18] | Decision tree | 0.70 | 0.68 | 0.72 | Yes | ＞1 y | ＞500 | Text | General |
|  | LR | 0.64 | 0.63 | 0.66 |  |  |  |  |  |
| Cella et al (2020) [20] | LASSO | 0.81 | 0.72 | 0.90 | Yes | ≤1 y | ≤500 | Sensor or  Multimodal | General |

*Abbreviation: AUC, Area Under The Curve; CI, Confidence Interval; RF, Random Forest; LR, Logistic Regression; SVM, Support Vector Machine; XGBoost, eXtreme Gradient Boosting; LightGBM, Light Gradient Boosting Machine; AdaBoost, Adaptive Boosting; KNN, K-nearest Neighbors; HCRNN, Hybrid-convolutional recurrent neural network; LASSO, Least Absolute Shrinkage and Selection Operator.*

*Several studies developed multiple models using the same underlying cohort; each row represents a model-level discrimination estimate.*

*Only the best-performing model from each study cohort was included in the primary meta-analysis to avoid double-counting of participants. The selected model was defined according to the original study authors’ designation of the best-performing model within that cohort.*

*All models from each study cohort were additionally included in the within-study weighted average meta-analysis.*

*Prespecified subgroup classifications were defined according to the prediction time window (≤1 y vs ＞1 y), sample size (≤500 vs ＞500 participants), data modality (text-based vs sensor-based or multimodal inputs), and population subgroup (general vs specific conditions).*

*AUC values and corresponding 95% confidence intervals are presented as originally reported.*

*
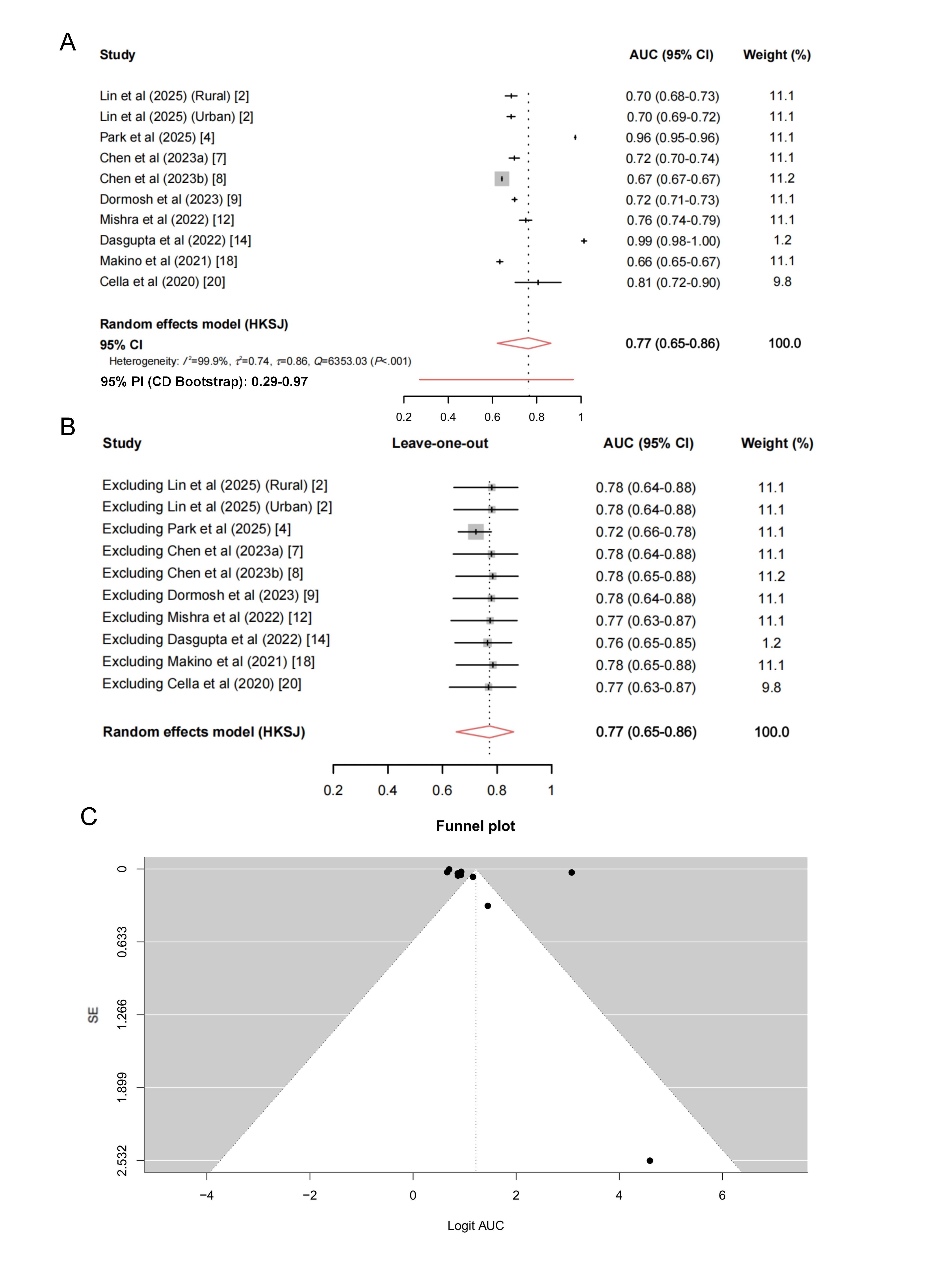
*

*Figure S2.* Random-effects meta-analysis of model discrimination performance using within-study average AUC estimates. (A) Forest plot presenting sample-level averaged AUCs, derived by aggregating multiple models trained on the same dataset. Individual study estimates with corresponding 95% CIs are shown alongside the pooled AUC estimated using the HKSJ method (back-transformed from the logit scale). Between-study heterogeneity is quantified using *I*^2^ , *τ*^2^, *τ* and *Q* statistic. Four types of PIs (HTS, HTS-HK, HTS-SJ, CD-bootstrap) are also reported in the pooled summary to illustrate the expected range of model performance in future populations. (B) Leave-one-out sensitivity analysis, in which the pooled AUC is recalculated after sequential exclusion of each study to assess the robustness of the overall estimate. (C) Funnel plot of logit-transformed sample-level AUC values to evaluate potential small-study effects.


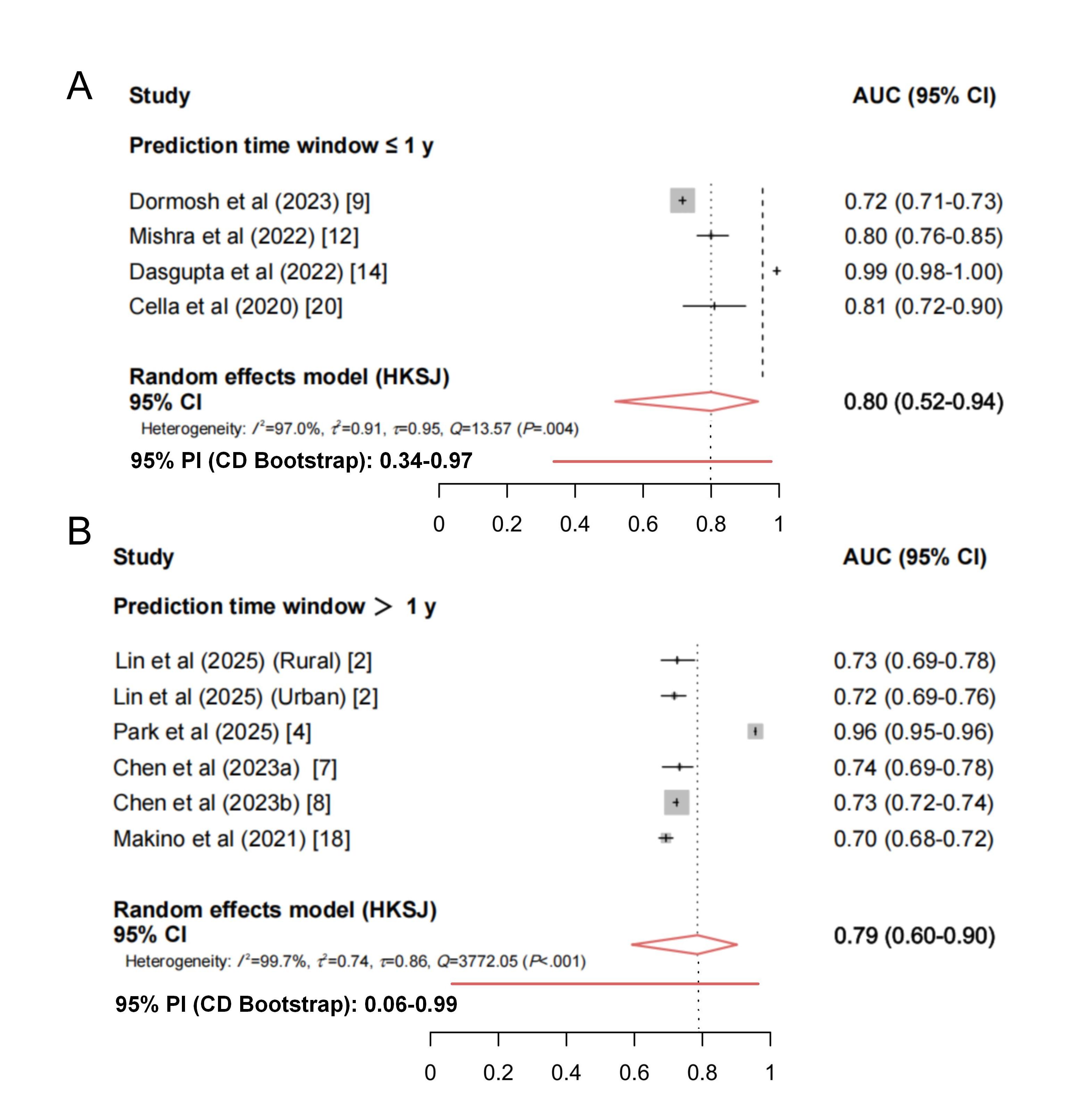


*Figure S3.* Subgroup meta-analysis of model discrimination by prediction time window (≤1 y vs ＞1 y) among 10 fall risk prediction models included in the meta-analysis. (A) Prediction time window ≤1 y. (B) Prediction time window ＞1 y.

Forest plots present sample-level AUCs with corresponding 95% CIs and subgroup-specific pooled AUCs estimated using the HKSJ random-effects model on the logit scale and back-transformed to the AUC scale. Between-study heterogeneity within each subgroup is quantified using *I*^2^ , *τ*^2^, *τ* and *Q* statistic. 95% PI (CD-bootstrap) is also reported to illustrate the expected dispersion of model discrimination in future populations. PIs estimated using alternative methods are similarly wide (prediction time window ≤1 y: HTS 0.40-0.94; HTS-HK 0.44-0.94; HTS-SJ 0.44-0.93; prediction time window ＞1 y: HTS 0.09-0.99; HTS-HK 0.22-0.98; HTS-SJ 0.22-0.98). Meta-regression showed no significant difference between prediction time window subgroups (test of moderators: *F*(1,8) = 0.004, *P* = 0.95).


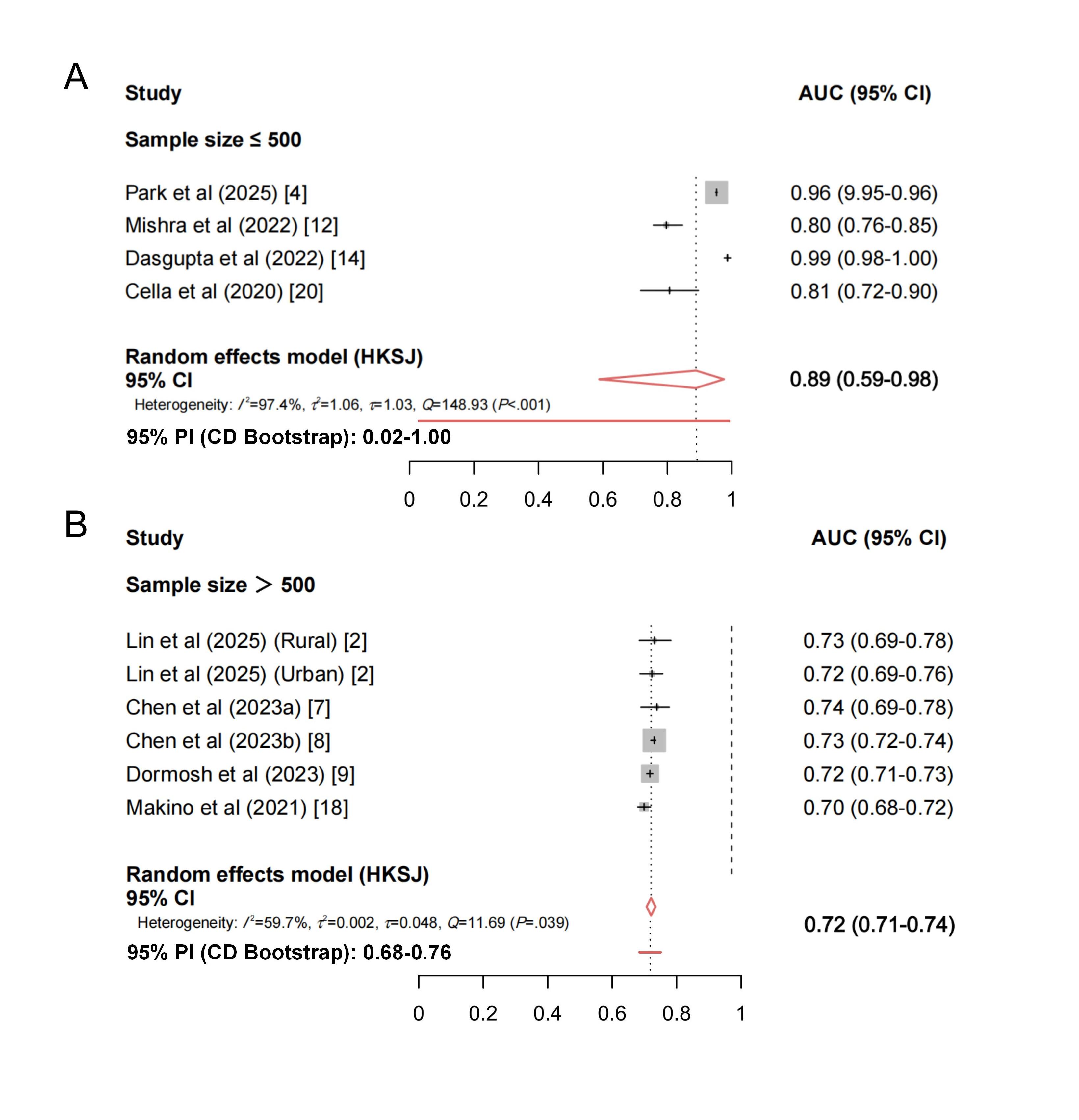


*Figure S4.* Subgroup meta-analysis of model discrimination by sample size (≤500 vs ＞500 participants) among 10 fall risk prediction models included in the meta-analysis. (A) Sample size ≤500. (B) Sample size ＞500.

Forest plots present sample-level AUCs with corresponding 95% CIs and subgroup-specific pooled AUCs estimated using the HKSJ random-effects model on the logit scale and back-transformed to the AUC scale. Between-study heterogeneity within each subgroup is quantified using *I*^2^ , *τ*^2^, *τ* and *Q* statistic. 95% PI (CD-bootstrap) is also reported to illustrate the expected dispersion of model discrimination in future populations. PIs estimated using alternative methods are similarly wide (sample size ≤500: HTS 0.02-1.00; HTS-HK 0.07-1.00; HTS-SJ 0.07-1.00; sample size ＞500: HTS 0.69-0.75; HTS-HK 0.69-0.75; HTS-SJ 0.69-0.75). Meta-regression indicated a statistically significant subgroup effect of sample size (test of moderators: *F*(1,8) = 8.75, *P* = 0.018), explaining approximately 42% of between-study heterogeneity (*R*^2^ = 42.03%). Higher discriminative performance was observed in models developed from smaller samples.


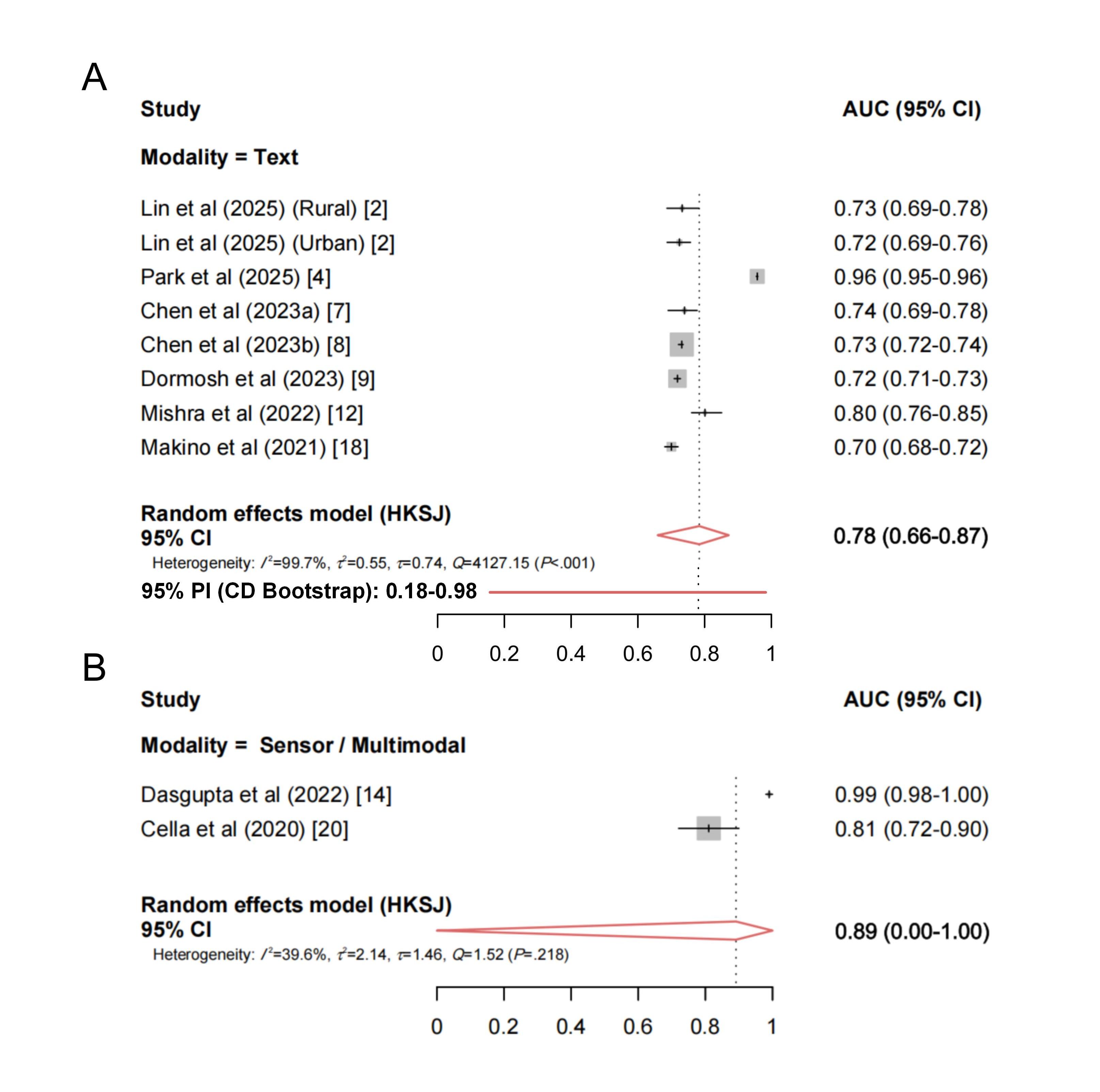


*Figure S5.* Subgroup meta-analysis of model discrimination by data modality (text-based vs sensor-based/multimodal inputs) among 10 fall risk prediction models included in the meta-analysis. (A) Text-based models. (B) Sensor-based or multimodal models.

Forest plots present sample-level AUCs with corresponding 95% CIs and subgroup-specific pooled AUCs estimated using the HKSJ random-effects model on the logit scale and back-transformed to the AUC scale. Between-study heterogeneity within each subgroup is quantified using *I*^2^ , *τ*^2^, *τ* and *Q* statistic. 95% PI (CD-bootstrap) is reported where estimable. PIs estimated using alternative methods are similarly wide (text-based models: HTS 0.23-0.98; HTS-HK 0.34-0.96; HTS-SJ 0.34-0.96). Meta-regression did not identify a significant difference between modality subgroups (test of moderators: *F*(1,8) = 0.33, *P* = 0.58).
*Note:* The sensor-based/multimodal subgroup included only two models; PIs could not be reliably estimated and are therefore not displayed.


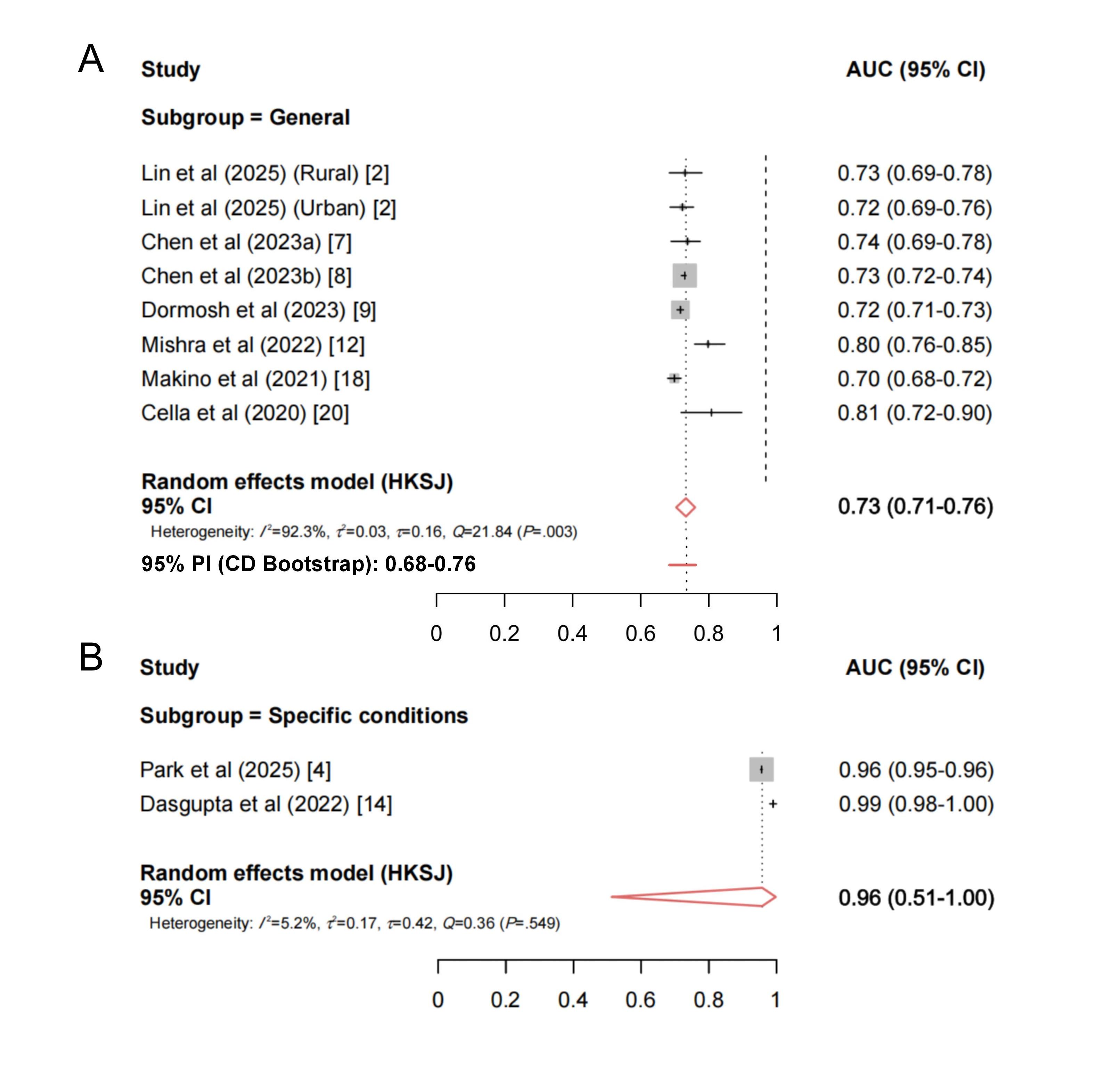


*Figure S6.* Subgroup meta-analysis of model discrimination by population subgroup (general vs specific conditions) among 10 fall risk prediction models included in the meta-analysis. (A) Subgroup = general. (B) Subgroup = specific conditions.

The forest plot presents sample-level AUCs with corresponding 95% CIs and subgroup-specific pooled AUCs estimated using the HKSJ random-effects model on the logit scale and back-transformed to the AUC scale. Between-study heterogeneity within each subgroup is quantified using *I*^2^ , *τ*^2^, *τ* and *Q* statistic. 95% PI (CD-bootstrap) is also reported where applicable to illustrate expected performance dispersion. PIs estimated using alternative methods are similarly wide (subgroup = general: HTS 0.69-0.75; HTS-HK 0.69-0.75; HTS-SJ 0.69-0.75). Meta-regression demonstrated a strong subgroup effect (test of moderators: *F*(1,8) = 115.97, *P* < 0.001), with population subgroup explaining approximately 87% of between-study heterogeneity (*R*^2^ = 86.99%). Models developed for specific populations showed substantially higher discrimination than those developed for general community-dwelling samples.
*Note:* The specific-population subgroup included only two models; PIs could not be reliably estimated and are therefore not shown.

*Table S4.* Comparison of the current review with key prior reviews on fall-related risk assessment or prediction.

| Review (y) | Inclusion criteria | Population | Setting | Outcome timing | Modality | Validation  types | Task focus | Whether  ML focus |
| --- | --- | --- | --- | --- | --- | --- | --- | --- |
| Machine learning-based prediction models for falls in hospitalized patients: A systematic review and meta-analysis (2025) [29] | (1) Original study reports; (2) Inpatient population; (3) ML algorithms designed to predict the risk of falls; (4) Reported at least one major outcome related to the prediction of fall risk; (5) Accessible full text; and (6) English studies. | Hospitalized patients | Hospital | During hospitalization; Short-term | Text | Not reported | Fall risk prediction models in hospitalized patients | Yes |
| The applications of artificial intelligence for assessing fall risk: Systematic review (2024) [30] | (1) Articles published in 2018-2023; (2) Studies applied AI methods; (3) AI should be applied to human data; and (4) The sample analyzed should consist of people with independent walking, with or without the use of external orthopedic devices. | People with independent walking | Heterogeneous | Cross-  sectional | Text or  Sensor or  Image | Not reported | Fall risk classification | Yes |
| A systematic review of fall prediction models for community-dwelling older adults comparison between models based on research cohorts and models based on routinely collected data (2024) [31] | (1) Community-dwelling older adults aged ≥60 ys, or from which the majority were aged >65 ys, or the mean age was >65 ys; and (2) Multivariable prediction models including ≥2 predictors for individual future fall risk estimation. | Community-dwelling older adults | Community | Longitudinal; Short-term and long-term | Text | Reported; Primarily internally validated | Short-term and long-term fall risk prediction models in community-dwelling older adults | No |
| Latest research trends in fall detection and prevention using  machine learning: A systematic review (2021) [32] | (1) Articles published after 2010; (2) The publishing venue is a Journal or Conference; (3) Studies used ML for fall detection or prevention; and (4) Studies used a detailed methodology and results. | No restrictions | Controlled/  lab-based settings or public datasets | Real-time/  Near-real-  time | Sensor | Not reported | Real-time fall detection and prevention | Yes |
| Research of fall detection and fall prevention technologies: A systematic review (2019) [33] | Fall detection systems based on sensing and analytical technologies. | No restrictions | Lab-based or simulation settings | Real-time/  Near-real-  time | Sensor | Not reported | Fall detection and fall prevention technologies | Yes |
| This systematic review | (1) Participants aged ≥60 ys at baseline; (2) Longitudinal studies; (3) Predictors measured at baseline; (4) ML models, including DL, for identifying community-dwelling older adults at high risk of future falls; (5) Assessment of fall-related outcomes; and (6) Publication in English with full-text available. | Community-dwelling older adults | Community | Longitudinal; Short-term and long-term | Text or  Sensor or  Image or Multimodal | Reported; Primarily internally validated | Short-term and long-term fall risk prediction models in community-dwelling older adults | Yes |

*Abbreviation: ML, Machine Learning; AI, Artificial Intelligence.*

*This table contrasts the scope and methodological focus of the current review with key prior reviews to clarify differences, highlight novelty, and prevent readers from conflating distinct tasks.*

| *Box S1*. First-fall prediction models: practical recommendations for development and reporting   - Define the target population explicitly (incident/first fall), and report baseline fall history. Where feasible, develop models in fall-naive cohorts or stratify by prior-fall status to avoid conflating incident and recurrent risk. - Specify the prediction horizon and outcome ascertainment window for first falls, and consider competing risks like death and institutionalization when follow-up is long, or event rates are low. - Use feature sets appropriate for first-fall settings: prioritize modifiable intrinsic risk (physical function, gait/balance measures, sensory impairment, comorbidity burden, frailty) and extrinsic/behavioral risk (medication burden/polypharmacy, activity exposure, environmental hazards) rather than relying on prior-fall history. - Tailor thresholds and risk stratification to intervention capacity: first-fall prevention often favors higher sensitivity at lower risk thresholds, with transparent reporting of threshold selection and decision-analytic utility, such as decision-curve analysis. - Report calibration and generalizability: evaluate calibration-in-the-large/slope, perform robust internal validation, and prioritize external validation across settings to assess generalizability. |
| --- |

*References*

1. Wan, R., D. Long, K. Wang, et al, *Predicting fall risk among older adults with sarcopenia in China using machine learning models: a six-y longitudinal study from CHARLS.* BMC Geriatrics, 2026.<http://doi.org/10.1186/s12877-026-06977-y>.

2. Lin, L., X. Liu, C. Cai, et al, *Urban-rural disparities in fall risk among older Chinese adults: insights from machine learning-based predictive models.* Front Public Health, 2025. **13**: p. 1597853.<http://doi.org/10.3389/fpubh.2025.1597853>.

3. Takeshita, Y., M. Onishi, H. Masuda, et al, *Machine Learning Prediction for Postdischarge Falls in Older Adults: A Multicenter Prospective Study.* J Am Med Dir Assoc, 2025. **26**(2): p. 105414.<http://doi.org/10.1016/j.jamda.2024.105414>.

4. Park, C., N. Kim, M. Kim, et al, *Advancing fall risk prediction in older adults with cognitive frailty: A machine learning approach using 2-y clinical data.* PLoS One, 2025. **20**(8): p. e0330672.<http://doi.org/10.1371/journal.pone.0330672>.

5. Liu, D., N.C. Binkley, A. Perez, et al, *CT image-based biomarkers acquired by AI-based algorithms for the opportunistic prediction of falls.* BJR|Open, 2023. **5**(1).<http://doi.org/10.1259/bjro.20230014>.

6. Silveira, H., J. Lima, J. Plácido, et al, *Dual-Task Performance, Balance and Aerobic Capacity as Predictors of Falls in Older Adults with Cardiovascular Disease: A Comparative Study.* Behavioral Sciences, 2023. **13**(6): p. 488.<http://doi.org/10.3390/bs13060488>.

7. Chen, X., L. He, K. Shi, et al, *Interpretable Machine Learning for Fall Prediction Among Older Adults in China.* American Journal of Preventive Medicine, 2023. **65**(4): p. 579-586.<http://doi.org/10.1016/j.amepre.2023.04.006>.

8. Chen, X., S. Lin, Y. Zheng, et al, *Long-term trajectories of depressive symptoms and machine learning techniques for fall prediction in older adults: Evidence from the China Health and Retirement Longitudinal Study (CHARLS).* Archives of Gerontology and Geriatrics, 2023. **111**: p. 105012.[http://doi.org/https://doi.org/10.1016/j.archger.2023.105012](http://doi.org/https:/doi.org/10.1016/j.archger.2023.105012).

9. Dormosh, N., M.C. Schut, M.W. Heymans, et al, *Predicting future falls in older people using natural language processing of general practitioners’ clinical notes.* Age and Ageing, 2023. **52**(4).<http://doi.org/10.1093/ageing/afad046>.

10. Ramsdale, E., M. Kunduru, L. Smith, et al, *Supervised learning applied to classifying fallers versus non-fallers among older adults with cancer.* Journal of Geriatric Oncology, 2023. **14**(4).<http://doi.org/10.1016/j.jgo.2023.101498>.

11. Ikeda, T., U. Cooray, M. Hariyama, et al, *An Interpretable Machine Learning Approach to Predict Fall Risk Among Community-Dwelling Older Adults: a Three-y Longitudinal Study.* Journal of General Internal Medicine, 2022. **37**(11): p. 2727-2735.<http://doi.org/10.1007/s11606-022-07394-8>.

12. Mishra, A.K., M. Skubic, L.A. Despins, et al, *Explainable Fall Risk Prediction in Older Adults Using Gait and Geriatric Assessments.* Frontiers in Digital Health, 2022. **4**.<http://doi.org/10.3389/fdgth.2022.869812>.

13. Kelly, D., J. Condell, J. Gillespie, et al, *Improved screening of fall risk using free-living based accelerometer data.* Journal of Biomedical Informatics, 2022. **131**: p. 104116.[http://doi.org/https://doi.org/10.1016/j.jbi.2022.104116](http://doi.org/https:/doi.org/10.1016/j.jbi.2022.104116).

14. Dasgupta, P., A. Frisch, J. Huber, et al, *Predicting falls within 3 months of emergency department discharge among community-dwelling older adults using self-report tools versus a brief functional assessment.* The American Journal of Emergency Medicine, 2022. **53**: p. 245-249.[http://doi.org/https://doi.org/10.1016/j.ajem.2021.12.071](http://doi.org/https:/doi.org/10.1016/j.ajem.2021.12.071).

15. Tang, Y.T. and R. Romero-Ortuno, *Using Explainable AI (XAI) for the Prediction of Falls in the Older Population.* Algorithms, 2022. **15**(10): p. 353.<http://doi.org/10.3390/a15100353>.

16. Cuaya-Simbro, G., A.I. Perez-Sanpablo, E.F. Morales, et al, *Comparing Machine Learning Methods to Improve Fall Risk Detection in Elderly with Osteoporosis from Balance Data.* J Healthc Eng, 2021. **2021**: p. 8697805.<http://doi.org/10.1155/2021/8697805>.

17. Omae, K., N. Kurita, T. Takeshima, et al, *Significance of Overactive Bladder as a Predictor of Falls in Community Dwelling Older Adults: 1-y Followup of the Sukagawa Study.* J Urol, 2021. **205**(1): p. 219-225.<http://doi.org/10.1097/JU.0000000000001344>.

18. Makino, K., S. Lee, S. Bae, et al, *Simplified Decision-Tree Algorithm to Predict Falls for Community-Dwelling Older Adults.* Journal of Clinical Medicine, 2021. **10**(21): p. 5184.<http://doi.org/10.3390/jcm10215184>.

19. Cuaya-Simbro, G., A.I. Perez Sanpablo, A. Muñoz-Meléndez, et al, *Comparison of Machine Learning Models to Predict Risk of Falling in Osteoporosis Elderly.* Foundations of Computing and Decision Sciences, 2020. **45**: p. 66-77.<http://doi.org/10.2478/fcds-2020-0005>.

20. Cella, A., A. De Luca, V. Squeri, et al, *Development and validation of a robotic multifactorial fall-risk predictive model: A one-y prospective study in community-dwelling older adults.* PLoS One, 2020. **15**(6): p. e0234904.<http://doi.org/10.1371/journal.pone.0234904>.

21. Silva, J., I. Sousa, and J.S. Cardoso, *Fusion of Clinical, Self-Reported, and Multisensor Data for Predicting Falls.* IEEE Journal of Biomedical and Health Informatics, 2020. **24**(1): p. 50-56.<http://doi.org/10.1109/JBHI.2019.2951230>.

22. Ye, C., J. Li, S. Hao, et al, *Identification of elders at higher risk for fall with statewide electronic health records and a machine learning algorithm.* International Journal of Medical Informatics, 2020. **137**: p. 104105.[http://doi.org/https://doi.org/10.1016/j.ijmedinf.2020.104105](http://doi.org/https:/doi.org/10.1016/j.ijmedinf.2020.104105).

23. Kuspinar, A., J.P. Hirdes, K. Berg, et al, *Development and validation of an algorithm to assess risk of first-time falling among home care clients.* BMC Geriatrics, 2019. **19**(1): p. 264.<http://doi.org/10.1186/s12877-019-1300-2>.

24. Gillain, S., M. Boutaayamou, C. Schwartz, et al, *Using supervised learning machine algorithm to identify future fallers based on gait patterns: A two-y longitudinal study.* Experimental Gerontology, 2019. **127**: p. 110730.[http://doi.org/https://doi.org/10.1016/j.exger.2019.110730](http://doi.org/https:/doi.org/10.1016/j.exger.2019.110730).

25. Howcroft, J., E.D. Lemaire, and J. Kofman, *Prospective elderly fall prediction by older-adult fall-risk modeling with feature selection.* Biomedical Signal Processing and Control, 2018. **43**: p. 320-328.[http://doi.org/https://doi.org/10.1016/j.bspc.2018.03.005](http://doi.org/https:/doi.org/10.1016/j.bspc.2018.03.005).

26. Deschamps, T., C.G. Le Goff, G. Berrut, et al, *A decision model to predict the risk of the first fall onset.* Experimental Gerontology, 2016. **81**: p. 51-55.[http://doi.org/https://doi.org/10.1016/j.exger.2016.04.016](http://doi.org/https:/doi.org/10.1016/j.exger.2016.04.016).

27. Marschollek, M., A. Rehwald, K.-H. Wolf, et al, *Sensors vs experts - A performance comparison of sensor-based fall risk assessment vs conventional assessment in a sample of geriatric patients.* BMC Medical Informatics and Decision Making, 2011. **11**(1): p. 48.<http://doi.org/10.1186/1472-6947-11-48>.

28. Bath, P.A., N. Pendleton, K. Morgan, et al, *New approach to risk determination: development of risk profile fornew falls among community-dwelling older people by use of a GeneticAlgorithm Neural Network (GANN).* The Journals of Gerontology: Series A, 2000. **55**(1): p. M17-M21.<http://doi.org/10.1093/gerona/55.1.M17>.

29. Xie, R., L. Shao, J. Pei, et al, *Machine learning-based prediction models for falls in hospitalized patients: A systematic review and meta-analysis.* Geriatr Nurs, 2025. **63**: p. 487-498.<http://doi.org/10.1016/j.gerinurse.2025.03.059>.

30. González-Castro, A., R. Leirós-Rodríguez, C. Prada-García, et al, *The Applications of Artificial Intelligence for Assessing Fall Risk: Systematic Review.* Journal of Medical Internet Research, 2024. **26**.[http://doi.org/https://doi.org/10.2196/54934](http://doi.org/https:/doi.org/10.2196/54934).

31. Dormosh, N., B. van de Loo, M.W. Heymans, et al, *A systematic review of fall prediction models for community-dwelling older adults: comparison between models based on research cohorts and models based on routinely collected data.* Age Ageing, 2024. **53**(7).<http://doi.org/10.1093/ageing/afae131>.

32. Usmani, S., A. Saboor, M. Haris, et al, *Latest Research Trends in Fall Detection and Prevention Using Machine Learning: A Systematic Review.* Sensors (Basel), 2021. **21**(15).<http://doi.org/10.3390/s21155134>.

33. Ren, L. and Y. Peng, *Research of Fall Detection and Fall Prevention Technologies: A Systematic Review.* IEEE Access, 2019. **7**: p. 77702-77722.<http://doi.org/10.1109/ACCESS.2019.2922708>.
